# Large Blooms of *Bacillales* (*Firmicutes*) Underlie the Response to Wetting of Cyanobacterial Biocrusts at Various Stages of Maturity

**DOI:** 10.1128/mBio.01366-16

**Published:** 2018-03-06

**Authors:** Ulas Karaoz, Estelle Couradeau, Ulisses Nunes da Rocha, Hsiao-Chien Lim, Trent Northen, Ferran Garcia-Pichel, Eoin L. Brodie

**Affiliations:** a Earth and Environmental Sciences, Lawrence Berkeley National Laboratory, Berkeley, California, USA; b School of Life Sciences, Arizona State University, Tempe, Arizona, USA; c Laboratoire Biogéosciences, Université de Bourgogne, Dijon, France; d Environmental Genomics and Systems Biology Division, Lawrence Berkeley National Laboratory, Berkeley, California, USA; e Department of Environmental Science, Policy and Management, University of California, Berkeley, California, USA; f Department of Environmental Microbiology, Helmholtz Centre for Environmental Research-UFZ, Leipzig, Germany; g Center for Fundamental and Applied Microbiomics, Biodesign Institute, Arizona State University, Tempe, Arizona, USA; CEH-Oxford

**Keywords:** *Firmicutes*, biological soil crust, carbon loss, ecological succession, ecosystem services, pulsed-activity event, resistance, stability

## Abstract

Biological soil crusts (biocrusts) account for a substantial portion of primary production in dryland ecosystems. They successionally mature to deliver a suite of ecosystem services, such as carbon sequestration, water retention and nutrient cycling, and climate regulation. Biocrust assemblages are extremely well adapted to survive desiccation and to rapidly take advantage of the periodic precipitation events typical of arid ecosystems. Here we focus on the wetting response of incipient cyanobacterial crusts as they mature from “light” to “dark.” We sampled a cyanobacterial biocrust chronosequence before (dry) and temporally following a controlled wetting event and used high-throughput 16S rRNA and rRNA gene sequencing to monitor the dynamics of microbial response. Overall, shorter-term changes in phylogenetic beta diversity attributable to periodic wetting were as large as those attributable to biocrust successional stage. Notably, more mature crusts showed significantly higher resistance to precipitation disturbance. A large bloom of a few taxa within the *Firmicutes*, primarily in the order *Bacillales*, emerged 18 h after wetting, while filamentous crust-forming cyanobacteria showed variable responses to wet-up across the successional gradient, with populations collapsing in less-developed light crusts but increasing in later-successional-stage dark crusts. Overall, the consistent *Bacillales* bloom accompanied by the variable collapse of pioneer cyanobacteria of the *Oscillatoriales* order across the successional gradient suggests that the strong response of few organisms to a hydration pulse with the mortality of the autotroph might have important implications for carbon (C) balance in semiarid ecosystems.

## IMPORTANCE

Desert biological soil crusts are terrestrial topsoil microbial communities common to arid regions that comprise 40% of Earth’s terrestrial surface. They successionally develop over years to decades to deliver a suite of ecosystem services of local and global significance. Ecosystem succession toward maturity has been associated with both resistance and resilience to disturbance. Recent work has shown that the impacts of both climate change and physical disturbance on biocrusts increase the potential for successional resetting. A larger proportion of biocrusts are expected to be at an early developmental stage, hence increasing susceptibility to changes in precipitation frequencies. Therefore, it is essential to characterize how biocrusts respond to wetting across early developmental stages. In this study, we document the wetting response of microbial communities from a biocrust chronosequence. Overall, our results suggest that the cumulative effects of altered precipitation frequencies on the stability of biocrusts will depend on biocrust maturity.

## INTRODUCTION

Drylands cover more than 40% of our planet’s continental area and host more than a third of the total human population (1). Ecosystem services provided within drylands, such as water and nutrient cycling, carbon sequestration, and climate regulation are therefore of global significance. Approximately half of arid and semiarid drylands are deprived of plants and are colonized by biological soil crust (biocrust), an assemblage of cyanobacterium-dominated bacterial communities, microalgae, mosses, and lichens in various proportions (2). Biocrusts colonize bare grounds and therefore constitute the first successional stage in the development of arid and semiarid zone ecosystems (3). The development of biocrusts itself constitutes a typical example of an ecologic succession, with an orderly and foreseeable process of species changes over time, a process that can be documented in terms of visual and molecular parameters (4). In fact, a common method to classify biocrusts is by the dominant organisms within the successional sequence: starting with least mature, “light” cyanobacterial crusts, followed by “dark” cyanobacterial crust, lichen crusts, and then the most mature moss crusts (2). The natural time scale for their formation ranges from years (for primary succession into cyanobacterial crusts) to decades (for late succession into moss crusts) (5). The final stage of this succession seems to be related to aridity, where harsher conditions prevent more developed stages (3).

Biocrusts contribute to dryland ecosystem functioning in many ways. These include fixing large amounts of carbon and atmospheric nitrogen (6), fixing (7) and exporting (8) nitrogen into the soil, releasing atmospheric reactive nitrogen (9), leaching a variety of metals and nonmetals into the soil (10), influencing local hydrology (11), stabilizing surface soil (11, 12), and impacting soil energy input through albedo control (4). The precipitation regime of regions colonized by biocrusts is characterized by short wet intervals followed by long dry periods; hence biocrusts are adapted to low and sporadic moisture adaptability as well as desiccation survival (13). Primary succession in biocrusts, especially in early cyanobacterial crusts, has received little attention despite their central role in desert ecosystems and conservation status of globally important drylands. A better understanding of the early stage biocrusts to wetting within the context of the primary ecologic succession will advance our understanding of dryland ecosystem functioning.

Previous studies on desiccation-hydration cycles of cyanobacterial biocrusts have focused on *Microcoleus vaginatus* spp., often the dominant member of early-successional-stage biocrust communities (14, 15), primarily for axenic cultures (14, 16), and very recently for wild populations of *M. vaginatus* (13). Nonetheless the responses of microbial populations from early successional biocrusts to hydration are less well known. High-throughput sequencing of 16S rRNA gene amplicons allows studies of the phylogenetic and taxonomic structures of microbial communities. In a recent study, biocrusts of a single successional stage collected from arid and hyperarid sites in the Negev Desert were hydrated with H_2_^18^O water and incubated under dark anoxic and light oxic conditions. Community dynamics were tracked by coupling RNA stable isotope probing (RNA-SIP) with pyrosequencing (17). This pioneering work showed that changes in operational taxonomic unit (OTU) richness and community composition happen rapidly within the first day.

In this study, we followed the hydration response of the bacterial community in a cyanobacterial biological soil crust chronosequence collected from a cold desert in the Colorado Plateau. The chronosequence corresponded to a space-for-time equivalent successional gradient from a cyanobacterium-dominated light crust to an early dark crust. A wetting experiment was performed in the laboratory simulating a rain event, controlling for temperature and light cycle. Biological soil crust (BSC) community composition was analyzed for dry crusts and followed at two time points within a day after wetting using Illumina MiSeq sequencing of 16S rRNA gene and 16S rRNA amplicons and quantitative PCR (qPCR).

## RESULTS

The study used dry biocrust samples from an apparent crust maturity gradient collected in a single site in Moab, UT, which were subjected to a brief period of hydration in the lab (see Fig. S1a in the supplemental material). The maturity gradient was validated post hoc elsewhere by measurement of concentrations for major pigments (4). We analyzed 16S rRNA gene (DNA community) and 16S rRNA (ribosomal community) before wetting (D [dry soil]) and at 18 h (WE [wetting early time point]) and 25.5 h (WL [wetting late time point]) after wetting using Illumina tag (iTag) sequencing of V4 region (Fig. S1b). These two time points, within approximately a day postwetting, corresponded to maximum surface CO_2_ and O_2_ gas flux at the crust surface based on a previous study using biocrusts from the same site (13). Total bacterial 16S copy numbers per gram of soil were assessed by qPCR. Since DNA and RNA take different snapshots of microbial communities, we distinguish between the bacterial communities based on rRNA gene and rRNA.

10.1128/mBio.01366-16.1 FIG S1  (A) Diagram depicting physical layout of the biocrust maturity gradient. Gray is used to denote the least mature biocrusts, and shades of brown are used to denote the remaining crusts, with darker shades corresponding to more mature crusts. (B) Sampling strategy of biocrust pre- and postwetting (D, dry, WE, wetting early time point, WL, wetting late time point). Download FIG S1, PDF file, 1.2 MB. Copyright © 2018 Karaoz et al. 2018 Karaoz et al. This content is distributed under the terms of the Creative Commons Attribution 4.0 International license.

Of the 90 nucleic acid samples (45 rRNA gene and 45 rRNA) subjected to sequencing, 88 yielded sufficient quantities of sequences to allow subsequent analysis (total, 8,546,455 overlapped read pairs; range, 26,416 to 180,120 sequences/sample for rRNA gene and 47,790 to 210,391 sequences/sample for rRNA; median, 75,741 sequences/sample for rRNA gene and 105,967 sequences/sample for rRNA). Overlapped reads from 88 samples were used to define 5,133 OTUs encompassing 90% of all the reads, a figure of diversity which is at least an order of magnitude lower than current estimates of soil bacterial diversity (18, 19). Good’s coverage estimates suggest that 98.5 to 99.8% of the OTU diversity was sampled at the current depth of sequencing.

### Ecologic succession of crust microbial communities along the maturity gradient.

**FIG 1:**
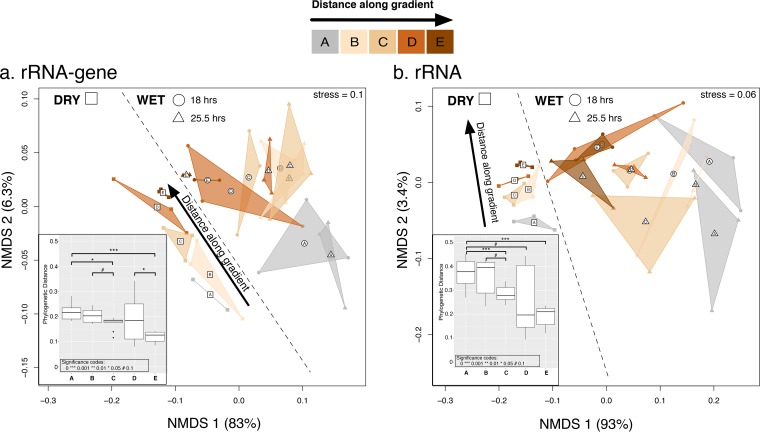
Nonmetric multidimensional scaling (NMDS) ordination of biocrust microbial communities, assessed by rRNA gene (a) and rRNA (b), based on weighted UniFrac phylogenetic distance metric. The percentage of total variation that can be explained by the distance along the successional gradient and sampling time point (D, dry; WE, wetting early time point at 18 h after wetting; and WL, wetting late time point at 25.5 h after wetting) is noted for the two ordination axes. Closed symbols denote individual samples, and their shapes correspond to different time points (rectangles, dry; circles, 18 h postwetting; triangles, 25.5 h postwetting). Colors denote the distance along the successional gradient, with darker colors corresponding to more mature crusts. Biological replicates are connected with solid lines; the resulting triangle is shaded by the color corresponding to the corresponding maturity level. The triangle centroid is marked with open symbols. Insets are box plots showing weighted UniFrac distances between communities from dry biocrusts and biocrusts sampled after wetting across the successional gradient. *P* values are for two-sided Wilcoxon tests.

To test and characterize the succession of microbial communities with the maturation of biocrusts, we computed phylogenetic distances (20) between microbial communities for all sample pairs and examined the results in a nonmetric multidimensional scaling (NMDS) ordination space (Fig. 1). Crust microbial communities showed clear successional patterns in which the communities diverged gradually with increasing distance along the gradient. These successional trajectories (marked with an arrow in Fig. 1) were qualitatively similar for dry and wet crusts and to a large degree followed the sampled gradient. Most noticeably, the magnitude of total variability in phylogenetic distances attributed to wetting was at least as large as the variability attributed to crust maturity (rRNA gene, 34 and 32%; rRNA, 53 and 21% for crust maturity and sampling time, respectively). Overall, the ordinations of samples with respect to successional gradient and wetting were to a large degree consistent for rRNA gene and rRNA (procrustes correlation = 0.81, significant at *P* < 0.001). Nevertheless, when between-sample dissimilarities based on rRNA gene were taken into account, additional variation in rRNA still significantly correlated with gradient and wetting (partial Mantel statistic, *r* = 0.16, *P* < 0.005).

In order to determine whether crust maturity was associated with changes in diversity and phylogenetic selection, we quantified how alpha diversity and phylogenetic dispersion changed along the successional gradient and postwetting. Calculation of traditional (Chao1 for richness, Pielou’s J for evenness, Shannon’s H′ for proportional diversity) and phylogenetic (Faith’s phylogenetic diversity [PD]) alpha diversity indexes showed that alpha diversity of dry biocrusts did not change significantly along the successional gradient (Spearman’s ρ test, α = 0.1) (see Fig. S2a to d in the supplemental material and Table S3 in the GitHub repository at https://github.com/ukaraoz/BiocrustSuccessionWetup). Overall, crust hydration did not have a significant and consistent effect on alpha diversity, with the trends appearing to be dependent on crust maturity (Fig. S2a to d). In contrast to alpha diversity, phylogenetic dispersion indexes, the net relatedness index (NRI), and the nearest-taxon index (NTI) revealed a significant shift to phylogenetically more-clustered communities along the chronosequence (NRI, Spearman’s ρ = 0.6; NTI, Spearman’s ρ = 0.53; *P* < 0.05 for NRI and NTI) (Fig. S2e and f). Overall, NTI was higher than NRI, indicating that clustering near the tips of the pool phylogeny was stronger compared to the clustering across the whole of the pool phylogeny. By 25.5 h, perturbation by wetting selected for even more clustered communities independent of the biocrust’s position within the chronosequence (analysis of variance [ANOVA] and Kruskal-Wallis contrasts, *P* < 0.05 for NTI and NRI) (Fig. S2e and f).

10.1128/mBio.01366-16.2 FIG S2  Diversity of microbial communities (by rRNA gene) associated with dry and wet biocrusts across the successional gradient as indicated by (a) OTU richness, (b) Shannon index, (c) Pielou’s evenness, (d) Faith’s phylogenetic diversity, (e) net relatedness index (NRI), and (f) nearest-taxon index (NTI). Download FIG S2, PDF file, 0.1 MB. Copyright © 2018 Karaoz et al. 2018 Karaoz et al. This content is distributed under the terms of the Creative Commons Attribution 4.0 International license.

To assess the source and significance of the variation in bacterial community structure along the successional gradient prewetting (D [dry]) and postwetting at two time points (18 h [WE] and 25.5 h [WL]), we used a permutational multivariate analysis of variance procedure (21). The compositions of crust microbial communities differed significantly with respect to sampling time when assessed by either 16S rRNA gene or rRNA (*P* < 0.001, ADONIS) (see Table S1 at https://github.com/ukaraoz/BiocrustSuccessionWetup). For both rRNA gene and rRNA, communities from dry crusts formed distinct clusters, while postwetting the communities from either time point were less discernible, suggesting that most of the postwetting response happened within 18 h. Nucleic acid type (rRNA gene/rRNA) was a significant factor structuring community composition, but its effect was smaller than time point and crust maturity (see Table S1 at the GitHub repository). Overall, the dispersion among replicates for dry and wet samples changed based on whether rRNA gene or rRNA sequences were used. When sampled using the rRNA gene, the dispersion in replicated communities did not change from dry to wet samples (dry, 0.097; 18 h, 0.108; 25.5 h, 0.1; Wilcoxon two-sided *P* value, >0.769). On the other hand, community sampling by rRNA was associated with significantly increased dispersion (dry, 0.077; 18 h, 0.14; 25.5 h, 0.136; Wilcoxon two-sided *P* value, <0.002).

### Mature biocrusts are more resistant to wetting.

To investigate whether the magnitude of microbial community shifts upon wetting depended on the crust maturity, we tested whether there were significant changes in the distribution of phylogenetic distances with increasing crust maturity (Fig. 1, insets). Nonmetric multidimensional scaling (NMDS) ordinations showed a clear separation between microbial communities from dry and wet biocrusts. The distances between communities from dry and wet biocrusts decreased significantly with increasing maturity, suggesting that more mature crusts were more resistant to wetting.

### Bacterial responses to wetting along the chronosequence.

Bacterial 16S rRNA gene and rRNA abundance, as measured by qPCR, were significantly higher (rRNA gene, *P* < 1E−4; rRNA, *P* < 0.005) in more mature biocrusts independent of whether they were sampled dry or postwetting (see Fig. S3 in the supplemental material and Table S2 at https://github.com/ukaraoz/BiocrustSuccessionWetup). A large proportion of this variability seemed to be driven by the low bacterial abundances in the least mature sandy soils (level A). When the analysis was repeated for crust levels B to E, the relationship between rRNA gene abundance and crust maturity was still consistent (rRNA gene, *P* < 0.001; rRNA, *P* < 0.05). No consistent relationship between wetting and bacterial abundance was detected across the chronosequence. Interestingly, the strengths of the relationships between crust maturity and bacterial rRNA gene and rRNA abundances were quite different (rRNA gene, partial η^2^ = 0.51; rRNA, partial η^2^ = 0.27). The abundance of bacterial rRNA gene increased nearly 6-fold across the crust gradient from B to E for dry crusts and 10-fold for wet crusts.

10.1128/mBio.01366-16.3 FIG S3  Abundance of bacterial 16S rRNA genes (closed circles) and rRNA (open circles) across the successional gradient prewetting (d) and postwetting (WE and WL). The broken lines show the mean for the biological replicates (*n =* 3). Download FIG S3, PDF file, 0.1 MB. Copyright © 2018 Karaoz et al. 2018 Karaoz et al. This content is distributed under the terms of the Creative Commons Attribution 4.0 International license.

### Taxonomic shifts through crust chronosequence wetting.

**FIG 2:**
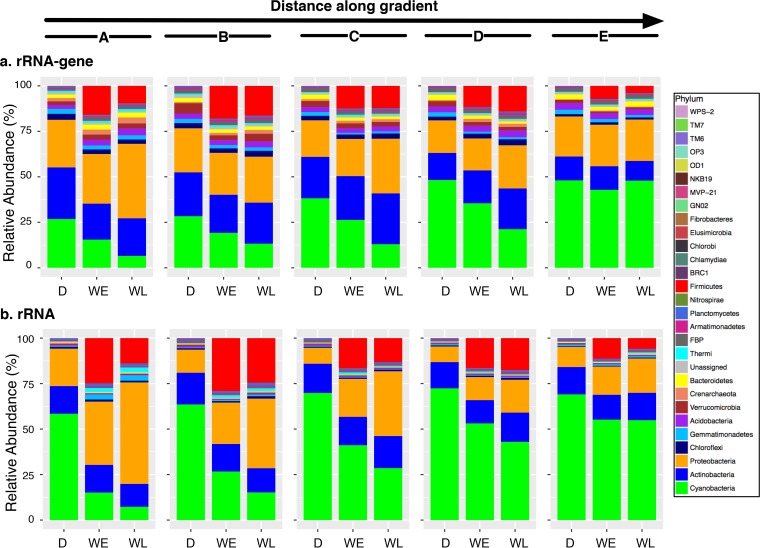
16S gene copy number corrected relative abundances of microbial phylogenetic groups summarized at the phylum level across the successional gradient prewetting (D, dry) and postwetting (WE, wetting early time point at 18 h after wetting; WL, wetting late time point at 25.5 h after wetting), based on 16S rRNA gene (a) and 16S rRNA (b). For each bar plot, the stacking order of phyla is fixed and as shown in the legend. A bloom of *Firmicutes* (red) after wetting is apparent within each subpanel.

To minimize the potential bias due to differences in 16S rRNA gene copy numbers across the detected phyla in our study (22), we normalized the relative abundances of OTUs defined by rRNA gene or rRNA using 16S gene copy number estimates based on taxonomic affiliations of these OTUs. This gave us estimates of organismal relative abundances (assuming a single genome copy/cell) (Fig. 2). All dry crusts, independent of their position within the successional gradient, were dominated by *Cyanobacteria*, *Actinobacteria*, and *Proteobacteria*, with OTUs associated with these three phyla accounting together for ~85% and ~95% of microbial communities sampled by rRNA gene and rRNA, respectively. Maturation of biocrusts corresponded to an increase in cyanobacterial taxa and a decrease in proteobacterial and actinobacterial taxa. We observed that wetting induced drastic phylum-level changes in the biocrusts’ microbial communities, primarily driven by a bloom of OTUs associated with *Firmicutes* (Fig. 2). When dry, *Firmicutes* accounted on average for 0.01% to 0.04% of microbial communities across the gradient, while 18 h after wetting, they increased to 7.2 to 18%.

### *Bacillales* underlie the response of biocrusts to wetting.

The drastic increase observed in relative abundance of the *Firmicutes* phylum after wetting prompted us (i) to ask whether this trend translated into significant increases in absolute abundance given the variations in total bacterial abundance and (ii) to evaluate response to wetting at finer phylogenetic resolution (i.e., lower taxonomic ranks). Organisms associated with the phylum *Firmicutes* significantly increased during wetting (ANOVA false-discovery rate [FDR]-adjusted *P* value, <0.05; Kruskal-Wallis contrast, *P* < 0.05) (see Fig. S4A in the supplemental material and Table S4 at https://github.com/ukaraoz/BiocrustSuccessionWetup), while changes in other dominant (≥1% of dry biocrusts) phyla were rather significant solely along the maturity gradient (Fig. S4A; see Table S4 at the GitHub repository). *Crenarchaeota* and *Nitrospirae* were the only two rare phyla (<1%) that increased significantly 18 h after wetting (ANOVA FDR-adjusted *P* value, <0.05; Kruskal-Wallis contrast, *P* < 0.05) (see Table S4 at the GitHub repository). Similar phylum-level trends were observed for rRNA associated with these organisms (Fig. S4B).

10.1128/mBio.01366-16.4 FIG S4  Abundance of organisms (A) and rRNA (B) associated with the dominant (≥1% of dry biocrusts) microbial phyla *Firmicutes*, *Cyanobacteria*, *Proteobacteria*, and *Actinobacteria* along the successional gradient for dry (D) and wet (WE and WL) biocrusts. Download FIG S4, PDF file, 0.3 MB. Copyright © 2018 Karaoz et al. 2018 Karaoz et al. This content is distributed under the terms of the Creative Commons Attribution 4.0 International license.

**FIG 3:**
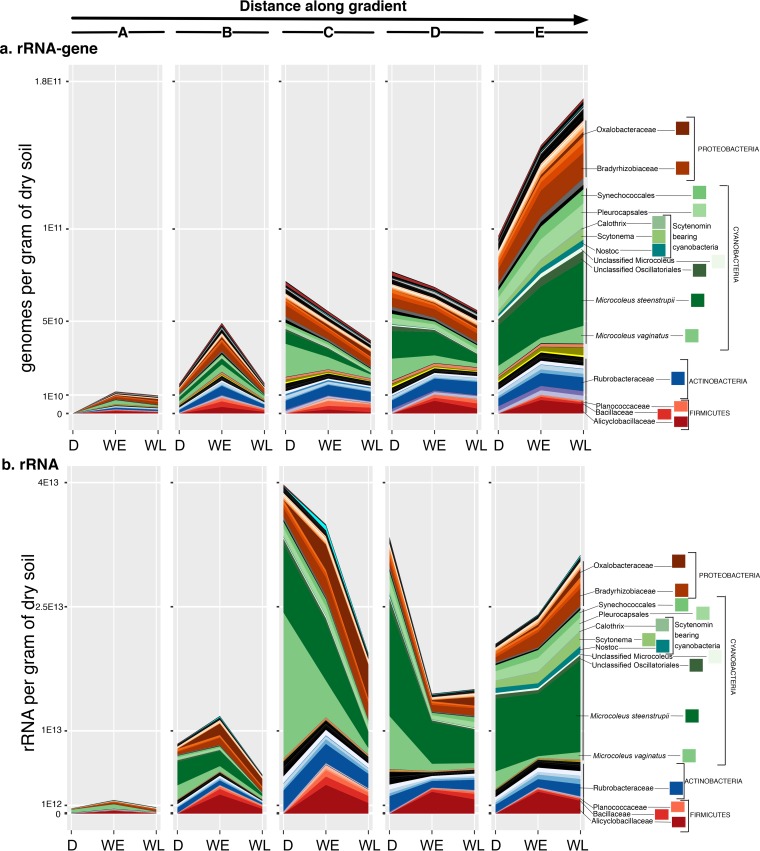
16S gene copy number corrected absolute abundances of microbial phylogenetic groups summarized at the family level across the successional gradient (*x-*axis labels are the same as in Fig. 2) by 16S rRNA gene (a) and 16S rRNA (b). Families associated with wetting-responsive OTUs (summarized in Table 1) are labeled on the right.

The abundances of organisms associated with several *Firmicutes* families, exclusively from the order *Bacillales*, surged significantly with wetting (ANOVA FDR-adjusted *P* value, <0.05; Kruskal-Wallis contrast, *P* < 0.05, based on rRNA gene) (Fig. 3). Among these, the families *Alicyclobacillaceae*, *Bacillaceae*, and *Planococcaceae* became by far the most abundant after wetting, together accounting for nearly 93.5% of all *Firmicutes* (see Fig. S5A and B in the supplemental material and Table S5 at https://github.com/ukaraoz/BiocrustSuccessionWetup). *Paenibacillaceae*, *Peptostreptococcaceae*, *Clostridiaceae*, and *Exiguobacteraceae* also increased significantly, 2 to 3 orders of magnitude, but they remained relatively rare (<0.5%) members of the microbial communities at the end of the wet period. The bloom of these *Firmicutes* families was accompanied by changes in *M. vaginatus* and *M. steenstrupii*, as well as scytenomin-bearing cyanobacteria (Fig. 3). Notably, these changes were not uniform across the successional gradient, with populations declining in earlier stages of the succession (maturity levels C and D) but increasing for the most mature biocrusts (level E) (Fig. S5A and B). Outside the *Firmicutes*, there were only two families that became abundant after wetting (≥1%) (Fig. 3). The archaeal family *Nitrososphaeraceae* increased significantly 18 h after wetting, while the proteobacterial *Oxalobacteraceae* family increased significantly throughout the wetting experiment (Fig. S5A and B; see Table S5 at the GitHub repository).

10.1128/mBio.01366-16.5 FIG S5  Abundance of organisms (A) and rRNA (B) associated with the microbial families and selected cyanobacterial phylotypes (a) *Alicyclobacillaceae*, (b) *Bacillaceae*, (c) *Planococcaceae*, (d) *Rubrobacteraceae*, (e) *Oxalobacteraceae*, (f) *Bradyrhizobiaceae*, (g) *Microcoleus vaginatus*, (h) *Microcoleus steenstrupii*, (i) *Nostoc*, (j) *Scytonema*, and (k) *Calothrix* along the successional gradient for dry (D) and wet (WE and WL) biocrusts. Download FIG S5, PDF file, 0.5 MB. Copyright © 2018 Karaoz et al. 2018 Karaoz et al. This content is distributed under the terms of the Creative Commons Attribution 4.0 International license.

### Co-occurrence patterns of community members pre- and postwetting.

To explore the associations between OTUs and further elucidate the importance of *Firmicutes* in biocrusts, we applied correlation-based network analysis by examining the co-occurrence patterns in dry and wet crusts separately. Co-occurrence networks indicate potential ecologic interactions between community members at various taxonomic resolutions and help in determining biologically relevant interactions for further study (23–25). We analyzed a total of 27 abundant OTUs (in either dry or wet biocrusts; see the method for filtering criteria described below). We estimated correlation values from compositional data across pre- and postwetting using SparCC (26), a tool previously benchmarked to generate putative interactions of high precision (27).

Co-occurrence patterns between OTUs were drastically different for dry and wet samples (see Fig. S10 in the supplemental material). In dry biocrusts, interactions were primarily restricted to members of *Microcoleus* and *Rhizobiales* (*Alphaproteobacteria*). Most notably, *M. vaginatus* (OTU 1) negatively correlated with several members of *M. steenstrupii*. Among *M. steenstrupii*, a single taxon represented by OTU 6, negatively correlated with three separate taxa from *Rhizobiales* order. Postwetting, of particular interest was the interaction partners of members of *Firmicutes*. Within *Firmicutes*, members of *Bacillales* represented by three OTUs were in the co-occurrence network. We detected a positive correlation between an *Alicyclobacillus* OTU (OTU 3692) and *M. vaginatus*. The same OTU had a negative correlation with a member of *M. steenstrupii*. OTU 5, of genus *Sporosarcina*, negatively correlated with two proteobacterial OTUs (OTU 12 of genus *Balneimonas* and OTU 58 of *Oxalobacteraceae*). Overall, in dry biocrusts, cyanobacterial interactions recapitulate the mutual exclusion between *M. vaginatus* and other *Microcoleus* species, including *M. steenstrupii* as we previously reported (4), while also pointing to possible competitive interactions between *M. steenstrupii* and members of *Rhizobiales*. In wet biocrusts, mutual exclusion of members of *Bacillales* with two proteobacterial taxa and *M. steenstrupii* is indicative of either competitive interactions or nonoverlapping niches between the primary producers, the blooming heterotrophs.

### A small number of OTUs explain most of the dynamics after wetting.

**FIG 4:**
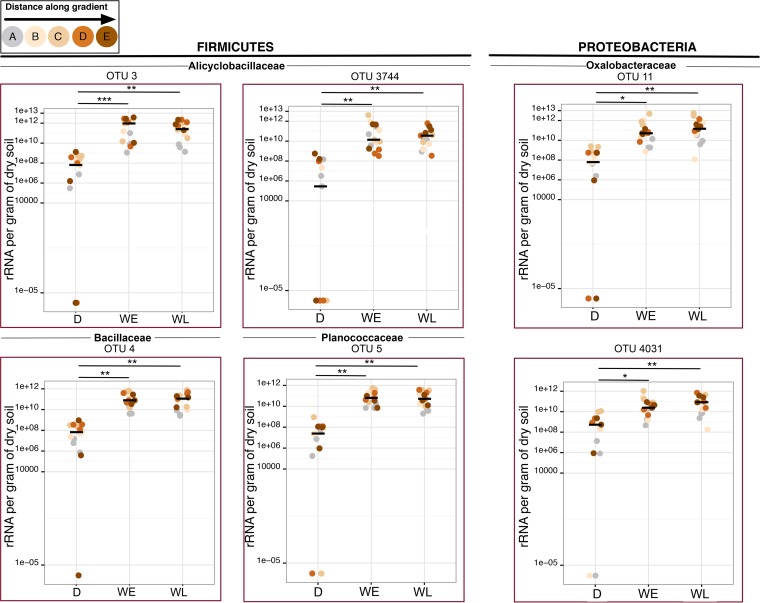
rRNA abundances of OTUs accounting for most of the wetting response and for which there was a significant wetting response. *x-*axis labels are the same as in Fig. 2 and 3, and colors denote the position within the chronosequence (color scheme same as in Fig. 1). Significant differences by Wilcoxon test are denoted by asterisks (two-sided *P* values): ***, *P* < 0.001; **, *P* < 0.01; *, *P* < 0.05.

To decipher the wetting response of biocrusts at the OTU level, we enumerated all the OTUs having at least 1% in relative abundance and increased in absolute abundance (absolute fold change of >1) postwetting at either time point. Given that all of the communities were highly uneven (see Fig. S6 in the supplemental material), this enumeration resulted in just 19 OTUs (see Table S6 at https://github.com/ukaraoz/BiocrustSuccessionWetup). The OTUs underlying the wetting response were consistent across communities defined by rRNA gene and rRNA, with 14 OTUs common to either molecular marker. Together, these OTUs explained most of the wetting response within their respective families (Table 1). Among these, for six OTUs, the increases in genome copies and rRNA were significant (Mann-Whitney two-tailed *P* < 0.05) (Fig. 4; see Fig. S7 in the supplemental material). Within three *Bacillales* families that significantly increased through wetting and became abundant, just five OTUs accounted for most this increase, jointly constituting almost 75% of all *Firmicutes* postwetting. Within the *Alicyclobacillaceae* family, wetting induced a more than 3-fold increase in the abundances of three *Alicyclobacillus* OTUs, which 18 h after wetting, together made up 6.2% of the microbial communities and 84.3% of the *Alicyclobacillaceae* family OTUs. The 16S rRNA V4 regions of the representative sequences for these three OTUs were 95 to 97% similar to that of the closest genomically sampled and clone relative available in GenBank, *Tumebacillus ginsengisoli* strain Gsoil 1105 (28), suggesting that these OTUs represent species of the *Tumebacillus* genus (see Fig. S8a in the supplemental material). For *Bacillaceae* (OTU 4) and *Planococcaceae* (OTU 5), a single OTU from each family dominated these families (53% and 74%, respectively) through wetting. The representative sequence for OTU 4 from the *Bacillaceae* family was identical to three separate *Bacillus* species previously isolated: *B. koreensis* (28), *B. korlensis* (29), and *B. beringensis* (30) (Fig. S8b). OTU 5 from *Planococcaceae* was identical to multiple isolated species of genus *Sporosarcina*, and its phylogenetic placement suggested that it is another species of this genus (Fig. S8c). All of the five *Firmicutes* OTUs were early responders (within 18 h), with no statistically significant changes detected between 18 and 25.5 h. In contrast, within proteobacterial family *Oxalobacteraceae*, the two significantly increasing OTUs (OTU 11 and OTU 4031) seemed to be responding more slowly. Their phylogenetic placement suggested that the closest isolates for these OTUs were representatives of *Massilia kyonggiensis*/*Massilia haematophila* (OTU 11, 99% identity) (31, 32) and *Telluria mixta* (OTU 4031, 98% identity) (33). Together, the analysis at the subfamily level clearly indicated that the observed biocrust wetting response was determined by a small number of phylogenetically clustered OTUs.

10.1128/mBio.01366-16.6 FIG S6  Rank abundance curves for biocrust microbial communities (D, dry [gray]; WE, 18 h after wetting [red]; WL, 25.5 h after wetting [blue]) showing that all of the biocrust communities sampled were highly uneven. The figure insets show the Pielou’s evenness indexes for biological replicates. Download FIG S6, PDF file, 0.3 MB. Copyright © 2018 Karaoz et al. 2018 Karaoz et al. This content is distributed under the terms of the Creative Commons Attribution 4.0 International license.

10.1128/mBio.01366-16.7 FIG S7  Genome abundances of 6 OTUs accounting for most of the wetting response and for which there was a significant wetting response. *x*-axis labels are the same as in Fig. 2 and 3; colors denote the position within the chronosequence (color scheme the same as in Fig. 1). Significant differences by Wilcoxon test are denoted by asterisks (two-sided *P* values): ***, *P* < 0.001; **, *P* < 0.01; *, *P* < 0.05. Download FIG S7, PDF file, 0.2 MB. Copyright © 2018 Karaoz et al. 2018 Karaoz et al. This content is distributed under the terms of the Creative Commons Attribution 4.0 International license.

10.1128/mBio.01366-16.8 FIG S8  16S rRNA trees showing the phylogenetic placement of wetting-responsive OTUs within their respective families inferred by maximum likelihood. (a) *Alicyclobacillaceae*. (b) *Bacillaceae*. (c) *Planococcaceae*. (d) *Rubrobacteraceae*. (e) *Bradyrhizobiaceae*. (f) *Oxalobacteraceae*. Bootstrap values (percentage of 500 bootstrap repetitions) are marked on internal nodes. OTUs are marked with red arrows for clarity. Download FIG S8, PDF file, 0.3 MB. Copyright © 2018 Karaoz et al. 2018 Karaoz et al. This content is distributed under the terms of the Creative Commons Attribution 4.0 International license.

**TABLE 1:** Wetting-responsive OTUs^a^

OTU no.	Phylum	Family	Genus or species	Relative abundance (%):								
				Of all bacteria (FC and/or rank)			Within phylum			Within family		
				D	WE	WL	D	WE	WL	D	WE	WL
2	*Cyanobacteria*	*Microcoleaceae*	*M. steenstrupii*	2.64 ± 2.26 (rank, 3/3,876)	2.88 ± 3.64 (FC, 1.28; rank, 2/3,957)	2.67 ± 2.82 (FC, 1.6; rank, 1/3,922)	6.95 ± 5.79	10.3 ± 8.2	13.11 ± 10.12	9.37 ± 9.66	17.21 ± 17.17	22 ± 17.65
9		*Pseudanabaenaceae*		1.32 ± 1.14 (rank, 8/3,876)	1.52 ± 1.34 (FC, 1.54; rank, 7/3,957)	1.42 ± 1.4 (FC, 1.43; rank, 6/3,922)	3.47 ± 3.02	5.47 ± 4.49	6.96 ± 7	66.14 ± 11.24	79.66 ± 23.39	76.7 ± 11.7
7		Unassigned		0.53 ± 1 (rank, 38/3,876)	2.25 ± 5.49 (FC, 5.73; rank, 8/3,957)	0.14 ± 0.19 (FC, 0.24; rank, 155/3,922)	1.39 ± 5.19	8.09 ± 12.75	0.68 ± 1.57			

3^b^	*Firmicutes*	*Alicyclobacillaceae*	*Tumebacillus*	0.0026 ± 0.0032 (rank, 1,382/3,876)	2.5 ± 2.77 (FC, 2,039; rank, 6/3,957)	1.68 ± 1.53 (FC, 1,307; rank, 7/3,922)	11.55 ± 11.6	19.19 ± 24.53	14.87 ± 17.8	50.98 ± 30.25	36.88 ± 40.05	40.4 ± 36.39
3692				0.001 ± 0.0016 (rank, 1,722/3,876)	2.1 ± 4.23 (FC, 2,171; rank, 10/3,957)	1.11 ± 1.9 (FC, 479; rank, 41/3,922)	4.44 ± 6.75	16.16 ± 20.6	9.91 ± 13	19.6 ± 15.96	31.04 ± 29	26.9 ± 30.28
3744^b^				0.0007 ± 0.00098 (rank, 2,071/3,876)	1.659 ± 2.3 (FC, 2,827; rank, 15/3,957)	0.75 ± 1.38 (FC, 1,049; rank, 34/3,922)	3.11 ± 6.08	12.72 ± 19.74	6.67 ± 13.14	13.72 ± 20.73	24.45 ± 35.36	18.1 ± 24.43
4^b^		*Bacillaceae*	*Bacillus*	0.0009 ± 0.001 (rank, 2,087/3,876)	1.67 ± 1.17 (FC, 3,649; rank, 12/3,957)	1.9 ± 1.62 (FC, 2,956; rank, 11/3,922)	4.02 ± 4.85	12.85 ± 8.56	16.82 ± 8.77	10.58 ± 11.33	53.13 ± 15.53	52.2 ± 16.81
5^b^		*Planococcaceae*		0.0027 ± 0.0027 (rank, 1,425/3,876)	1.66 ± 1.18 (FC, 1,109; rank, 13/3,957)	1.9 ± 1.64 (FC, 921; rank, 12/3,922)	12 ± 6.71	12.71 ± 11.73	16.85 ± 8.06	67.5 ± 23.51	74.11 ± 18.23	82.7 ± 16.99

8	*Actinobacteria*	*Rubrobacteraceae*	*Rubrobacter*	3.08 ± 0.87 (rank, 5/3,876)	3.53 ± 1.4 (FC, 1.39; rank, 5/3,957)	3.93 ± 1.3 (FC, 1.29; rank, 5/3,922)	14.91 ± 4	18.45 ± 4	18.86 ± 4.14	46.48 ± 8.98	40.5 ± 8.48	41.7 ± 7.32

12	*Proteobacteria*	*Bradyrhizobiaceae*	*Salinarimonas*	3.85 ± 1.09 (rank, 4/3,876)	3.86 ± 1.42 (FC, 1.31; rank, 3/3,957)	4.51 ± 1.67 (FC, 1.19; rank, 3/3,922)	17.41 ± 3.61	17.4 ± 4.31	15.81 ± 3.3	46.13 ± 5.72	44.63 ± 5.86	44.8 ± 4.08
15				1.71 ± 0.49 (rank, 7/3,876)	1.57 ± 0.47 (FC, 1.18; rank, 9/3,957)	1.54 ± 0.48 (FC, 1.02; rank, 9/3,922)	7.75 ± 1.91	7.06 ± 2.1	5.41 ± 2.26	20.53 ± 5.52	18.11 ± 5.34	15.3 ± 5.59
57				0.92 ± 0.34 (rank, 20/3,876)	1.25 ± 0.45 (FC, 2.04; rank, 17/3,957)	1.58 ± 0.69 (FC, 1.69; rank, 17/3,922)	4.19 ± 1.2	5.63 ± 1.53	5.55 ± 1.87	11 ± 2.57	14.45 ± 2.83	15.7 ± 3.55
11^b^		*Oxalobacteraceae*	*Massilia*	0.0124 ± 0.017 (rank, 609/3,876)	0.6 ± 0.7 (FC, 48.6; rank, 36/3,957)	2 ± 1.74 (FC, 123.9; rank, 14/3,922)	0.056 ± 0.093	2.71 ± 3.15	7.08 ± 6.04	10.36 ± 8.3	29.86 ± 17.8	37.8 ± 23
4031^b^			*Telluria*	0.05 ± 0.11 (rank, 278/3,876)	0.38 ± 0.38 (FC, 7.97; rank, 65/3,957)	1.04 ± 1.2 (FC, 16.24; rank, 29/3,922)	0.22 ± 0.64	1.74 ± 1.62	3.65 ± 3.32	42.14 ± 22.44	19.16 ± 10.7	19.5 ± 10.4

^a^ The table lists 14 OTUs making up at least 1% of relative abundance and increased in absolute abundance (absolute fold change [FC] of >1) postwetting at either time point. For each OTU, their mean relative abundance within all bacteria and their respective phylum/family prewetting (dry) and postwetting are given. The table also lists fold changes in genome abundances with respect to prewetting and changes in their rank.

^b^ OTUs that showed significant increase in genome copies postwetting (*P* < 0.05 by Mann-Whitney two-tailed test).

## DISCUSSION

The dynamics of biocrust resuscitation from dormancy upon wetting has previously been studied with respect to initiation of photosynthesis and respiration (34), transcriptional response of *M. vaginatus* (13), and development of metabolically active bacterial communities under light oxygenic and dark anoxic conditions from biocrusts in the Negev Desert, Israel (17). Our recent work has suggested that the process of crust maturation has consequences on the cyanobacterial community with the replacement of the main “architect” species *M. vaginatus* with *M. steenstrupii*. Moreover, crust darkening should have generalized effects on the crust system because these community changes are accompanied by increases in the surface temperature (4, 35). Increased precipitation frequencies predicted under climate change scenarios make biocrusts sensitive if not in danger of disappearance. Though it has become clear that simulated rain events induce large changes in microbial communities within a day (17), how a gradient of light to dark crust answers these wetting events is unknown.

Our study reports how the microbial populations in developing cyanobacterial biocrusts respond to sustained wetting. Using high-throughput sequencing of the 16S phylogenetic marker and a replicated design, we quantified phylogenetic succession of biocrust microbial communities with respect to short- and long-term factors. Strikingly, our data show that the effect of wetting (a recurring but sizable disturbance operating within a day) on microbial community structure is as large as that of ecologic succession (a predictable and orderly process operating within decades) despite the wildly different time scales through which these processes are occurring.

We observed that the more mature biocrusts were, the more their associated microbial communities were resistant to wetting. Despite this overall increase in community stability to wetting, the response of the cyanobacterial populations to wetting was not uniform across the maturity gradient. For example, in the case of the most mature crusts, populations of “architect” cyanobacteria (*Microcoleus* spp.) did not experience population collapse, unlike those from less mature crusts. Along the same gradient levels, there was a concurrent increase in scytenomin-bearing species of order *Nostocales*. Altogether, this apparent interaction between architect and scytenomin-producing species of cyanobacteria upon wetting might be a reflection of higher levels of ultraviolet A (UVA)-induced protein and DNA damage in less mature crusts eventually hindering waking of architect cyanobacteria upon wetting.

Biocrust taxa were phylogenetically clustered (by both NTI and NRI) across all successional stages, suggesting that these bacterial taxa share ecologic traits (i.e., tolerance to desiccation and UV stress and rapid wetting response) that are phylogenetically conserved (36). A much stronger clustering was apparent when NTI was used as a phylogenetic relatedness index, compared with the use of NRI, and there was an apparent increase in clustering by either index. Since NRI quantifies deeper divergences in the phylogeny (i.e., tree-wide clustering) than NTI (i.e., tip-level clustering), our data suggest recent diversification in evolutionary time scales. It is well established that phylogenetic relatedness of microorganisms does not often posit conservation of ecologic traits (37); nevertheless, phylogenetic conservatism has been documented for certain ecologic traits, such as habitat preference and soil moisture optimum (38–40). Microbial strategies relevant to biocrust maturation such as desiccation tolerance and quick response to wetting likely necessitate coordination of many physiological traits. There has been some recent evidence from Mediterranean ecosystems supporting their deep evolutionary origins (40). While our data from biocrusts support phylogenetic coherence of these traits, it also suggests that they might be a result of more recent evolutionary adaptation.

We found that a significant and unexpectedly large bloom of few phylogenetically constrained soil heterotrophs from the *Bacillales* order follows biocrust wetting. Members of *Firmicutes* phylum and in particular of the genus *Bacillus* are well represented in desert sand (41–43), and they may be relatively more abundant in desert soils compared to other biomes (44). In previous surveys of cryptogamic covers, they were not consistently detected as abundant members of biocrust microbial communities. Studying vertical stratification of biocrusts from Colorado Plateau, Garcia-Pichel et al. did plate counts of aerobic sporeformers and detected *Bacillales* populations by sequencing of the corresponding denaturing gradient gel electrophoresis (DGEE) bands (45). Among multiple targeted metagenomic surveys of biocrusts from hot and cold deserts (46–48), only one study reported detection of *Firmicutes* in subsurface soil (1- to 2-cm depth) (49). The moisture content of the soils when nucleic acids were extracted could explain the seeming absence of this group in some of these previous surveys. *Firmicutes* could have been bloomed to large proportions when wet for a few days but not been detected after a period of drought, indicating a significant mortality for this group. In a recent study, using H_2_O-SIP RNA, Angel et al. reported that in arid crusts from the Negev Desert, postwetting, bacteria of the *Bacillales* order grew (from <1% when dry) to constitute nearly half of the active community within a day (17). These crusts had a surprisingly small fractional representation of *Cyanobacteria* (~1.9%), while in our study some 25 and 65% of microbial communities were cyanobacterial by DNA and RNA, respectively. This large discrepancy might be due to a bias of H_2_O-SIP RNA against *Cyanobacteria* or a cyanobacterial rRNA content that is below its limit of detection.

Surprisingly, a few OTUs accounted for a large proportion of the wetting response. For instance, just five OTUs accounted for some 75 and 35% of the quick wetting response within *Firmicutes* and *Proteobacteria*, respectively. Several common traits of the closest relatives of these taxa are apparent. Within *Firmicutes*, all of the previously identified isolates are soil-dwelling aerobic sporeformers (29, 30, 50, 51). In the case of proteobacterial taxa, they were root or soil associated, and in the case of *Telluria* species, they carried an ability to degrade a range of complex carbohydrates, with a preference for carbohydrates and tricarboxylic acid (TCA) intermediates (31, 33). Overall, these traits are all consistent with broad metabolic capability and a lifestyle evolved for slow growth in a nutrient-limited environment. Further elucidation of the fitness traits of these organisms and how they relate to soil quality through organic matter decomposition, carbon cycling, and soil aggregate formation will require genome-centric metagenomic studies and isolation efforts.

Relative abundance of rRNA depends on growth, starvation, death, and gene regulation. In our study, the variation attributed to the type of measurement (rRNA gene or rRNA) was significant but much smaller than the two other factors, crust maturity and wetting. Among abundant OTUs (>0.5% pre- or postwetting), significant overrepresentation of rRNA relative to rRNA gene was rare (see Fig. S9 in the supplemental material). This is in contrast to other studies of soil community composition concluding that RNA and DNA tend to be highly divergent (using a threshold of 1), which has been interpreted as a result of prevalent dormancy (52–54). Notably, none of the wetting-responsive OTUs had their rRNA statistically enriched relative to their genome copies. Taxon-specific variables, such as the strength and nature of the relationship between rRNA and rRNA gene concentrations, and cell size render assessment of the significance of RNA/DNA ratios difficult. Nevertheless, the observation that just two of the abundant OTUs had significantly more ribosomes per cell prewetting suggests resuscitation strategies other than conservation of ribosomes in the cell, such as rapid exit from sporulation or the ability to consume labile C or to extracellularly hydrolyze complex macromolecular C (55). Future genome-centric metagenomics and metatranscriptomics efforts will reveal genome signatures of these adaptive strategies.

10.1128/mBio.01366-16.9 FIG S9  RNA/DNA ratio of abundant (>1% relative abundance) OTUs. Significance was conducted with Wald test based on a negative binomial generalized linear model (GLM) fit (DEseq2) for the D (dry), WE (18 h after wetting), and WL (25.5 h after wetting) data sets separately, combining data across all maturity levels. Each circle represents an OTU, with size scaled based on the average relative abundance. OTUs that were significantly represented between rRNA and rRNA gene (FDR-corrected two-sided Wilcoxon *P* value < 0.01) are represented by filled circles. Download FIG S9, PDF file, 0.1 MB. Copyright © 2018 Karaoz et al. 2018 Karaoz et al. This content is distributed under the terms of the Creative Commons Attribution 4.0 International license.

10.1128/mBio.01366-16.10 FIG S10  Network of co-occurring OTUs (represented by nodes) in dry and wet (25.5 h after wetting) biocrusts based on SparCC correlation analysis. OTUs with absolute values of ≥0.3 are connected with solid lines, where the color indicates the sign of the interaction (blue, ≥0.3; red, ≤−0.3). Node size is proportional to the mean relative abundance in dry/wet samples. Nodes are colored based on the taxonomic affiliation of the OTU. Download FIG S10, PDF file, 0.3 MB. Copyright © 2018 Karaoz et al. 2018 Karaoz et al. This content is distributed under the terms of the Creative Commons Attribution 4.0 International license.

The strong response of few OTUs to a hydration pulse might have important implications for carbon (C) balance in semiarid ecosystems. It is well established that together with seasonal temperature, pulses of moisture availability are one of the main drivers of biological activity in biocrusts (35, 56). Consequently, biocrusts are highly susceptible to small fluctuations in precipitation regimes, as predicted by climate change models. In these pulse-driven systems, the magnitude of the rainfall events is an important determinant of the active components and their degree of activity (57, 58). Specifically, respiration and photosynthesis are turned on at different moisture levels (59). If increased pulse wetting predicted under climate change is not enough to reach the photosynthetic compensation point, it may result in the mortality of the autotroph (60) in the short term and C loss from the system over longer periods. The consistent *Bacillales* bloom accompanied by the variable collapse of the *Microcoleus* we documented across the successional gradient suggests that the cumulative effects of increased precipitation frequencies on C cycling will depend on crust maturity.

## MATERIALS AND METHODS

### Field sampling.

Field sampling was performed as previously described by Couradeau et al. (4). Briefly, biological soil crust samples were collected in the Green Butte Site near Canyonlands National Park (38°42′53.9″N, 109°41′34.6″W, Moab, UT). Biocrust cores were sampled using petri dishes (6-cm diameter, 1-cm depth) distributed on a 5-by-5 grid, covering a total square field of 50 cm by 50 cm (Fig. S1a). The five lines of the grid (denoted A, B, C, D, and E) correspond to increasing levels of crust colonization as determined in the field by the increase of dark pigmentation of the soil surface (A being the lowest level of colonization and E the highest). For each grid line, five petri dishes were collected, and three of those were randomly picked as biological replicates for this study (a total of 15 petri dishes). Samples were transported air dried and maintained in the dark under an atmosphere in equilibrium with LiCl desiccant until experimentation.

### Biological soil crust wet-up.

After collection of an initial dry subsample from each of the 15 petri dishes, each sample was subjected to a simulated rainfall event, receiving 10 ml of Milli-Q water (Merck Millipore, MA). A day/night cycle was simulated using a 400-W lamp source in a windowless room. While wet, the samples were submitted to 11 h of daylight at ~600 µmol photons m^−2^ s^−1^, 2 h at ~24 µmol photons m^−2^ s^−1^ simulating the dusk, 9 h in the dark, and 2 h of dawn at ~24 µmol photons m^−2^ s^−1^. These conditions mimicked a typical overcast summer day in Moab. All of the petri dishes were resampled 18 h (at night) and 25.5 h (at daytime) after the wetting event (Fig. S1b). All samples were immediately frozen in liquid nitrogen and stored at −80°C until nucleic acid extraction.

### Nucleic acid extraction and cDNA synthesis.

For nucleic acid extraction, we implemented a well-established extraction protocol that has been successfully used by us and others to effectively extract nucleic acids (including from bacterial and fungal sporeformers) from complex soils (61–65). This DNA/RNA coextraction protocol consists of combinations of bead beating, detergents, enzymatic lysis, and organic solvent extraction.

At each time point, samples of coherent crust (the top few millimeters) were taken and flash frozen in liquid nitrogen, while a separate sample was used to measure water content. During wetting, one-third biological soil crust samples in petri dishes were collected and homogenized per time point per gradient level per biological replicate using a flamed spatula and snap-frozen using liquid nitrogen. Prior to nucleic acid extraction, the biological soil crust samples were stored at −80°C. Phenol-chloroform-isoamyl alcohol (0.5 ml at 25:24:1) (Sigma-Aldrich, St. Louis, MO) was added to 0.5 g of biocrust sample in a 2-ml lysing matrix E tube (MP Biomedicals, Solon, OH), followed by addition of 0.5 ml of cetyltrimethylammonium bromide (CTAB) buffer (5% CTAB, 0.25 M phosphate buffer [pH 8.0], 0.3 M NaCl), and 50 μl of 0.1 M aluminum ammonium sulfate. The samples were beaten at 5.5 m/s for 30 s in a FastPrep-24 instrument (MP Biomedicals, Solon, OH), and centrifuged at 16,000 × *g* for 5 min at 4°C. The aqueous phase was transferred to MaxTract high-density 2-ml tubes (Qiagen, Valencia, CA). A second round of extraction with 0.5 ml CTAB buffer, and beating was performed. An equal volume of chloroform was added to each MaxTract high-density tube and centrifuged at 16,000 × *g* for 5 min at 4°C. The aqueous phase was transferred to a new 2-ml Eppendorf tube, and the nucleic acids were precipitated overnight with 2 volumes of 30% (wt/vol) polyethylene glycol 6000 and 1.6 M NaCl. The crude pellets of DNA and RNA were resuspended in 30 μl of diethyl pyrocarbonate (DEPC)-treated water and stored at −80°C. Fully automated separation of DNA and RNA was achieved using the AllPrep DNA/RNA minikit (Qiagen, Valencia, CA) and the QIAcube (Qiagen, Valencia, CA) following the manufacturer’s instructions. Total extracted RNA was reverse transcribed to cDNA using the NEBNext mRNA second strand synthesis module (New England Biolabs, Ipswich, MA) with random hexamers.

### Illumina sequencing of 16S rRNA gene and rRNA amplicons.

The V4 region of the SSU rRNA gene was amplified from DNA and cDNA templates using the primer pair 515F (5′-GTGCCAGCMGCCGCGGTAA-3′) and 806R (5′-GGACTACHVGGGTWTCTAAT-3′) as described by Couradeau et al. (4).

### DNA and cDNA qPCR.

Quantitative kinetic real-time PCRs (qPCRs) were performed using MyiQ reverse transcriptase PCR (RT-PCR; Bio-Rad, Hercules, CA). All qPCRs were run in triplicate with the general SSU rRNA gene primer set 338F (5′-ACTCCTACGGGAGGCAGCAG-3′) and 518R (5′-GTATTACCGCGGCTGCTGG-3′). PCRs were done using SsoFast EvaGreen Supermix (Bio-Rad, Hercules, CA) under the following conditions: initial denaturation for 2 min at 98°C followed by 40 cycles of denaturation at 95°C for 10 s and annealing at 55°C for 30 s. Melting curve analyses were performed from 55 to 95°C at 0.5°C s^−1^. Nucleic acid concentrations were determined with Qubit (Life Technologies, Inc., NY).

### Calculations of DNA and cDNA copies per gram of dry soil.

Soil material was carefully scraped from each frozen sample using a sterile spatula and deposited onto a scale until exactly 0.5 g was attained. This material was then used to extract nucleic acids. The water content of each sample was determined separately on corresponding parallel samples taken contemporaneously during the experiment. Soil moisture was determined using the following equation: *W* = ((*M*_1_ – *M*_2_)/(*M*_1_ – *M*_3_)) × 100. Where *W* is the percentage of water content, *M*_1_ is the wet soil plus container mass, *M*_2_ is the dry soil (105°C overnight) plus container mass, and *M*_3_ is the container mass. The number of DNA/cDNA copies per gram of dry soil was calculated by dividing the number of DNA/cDNA copies per reaction by grams of dry soil per reaction.

### Analysis of 16S rRNA gene and rRNA iTag sequences.

Pairs of forward and reverse reads were aligned using the usearch (v7.0.1090) (66) fastq_mergepairs command with -fastq_maxdiffs set to 3. The aligned reads were quality filtered with usearch fastq_filter command with -fastq_trunclen=250 bp., -fastq_maxee=0.1. Reads from all samples (rRNA gene and rRNA) were collected in a single fasta file, and singletons were removed using the usearch sortbysize command (minsize=2). The resulting sequences were used for OTU clustering with the uparse pipeline (67), setting the OTU cutoff threshold to 97%. Chimeric sequences were filtered with uchime (68) (usearch -uchime_ref) using the ChimeraSlayer (69) reference database downloaded from http://drive5.com/uchime/gold.fa. OTU abundances across individual samples were calculated by mapping chimera-filtered OTUs against the quality-filtered reads using the usearch usearch_global command (-strand plus -id 0.97).

### Taxonomic assignment and phylogeny inference.

We used SILVA reference files (release 123) available from mothur (70) for taxonomic classification and phylogeny inference. Taxonomy was assigned with a naive Bayes classifier (classify.seqs command in mothur) trained with SILVA full-length sequences and taxonomic references, except for cyanobacteria, for which manual taxonomic classification was performed following the most recent classification system outlined by Komarek et al. (71) as follows. The 16S rRNA V4 sequences and the cyanobacterial representative OTU sequences from the present study were placed into a phylogenetic tree. For each cyanobacterial OTU, the taxonomic name from the closest genome relative that corresponds to at least 97% sequence similarity was assigned.

For phylogeny inference, first the region bounded by primers was determined using mothur (v.1.37.0), and the Silva SEED alignment was sliced (start=10264, end=25298). Representative sequences for each OTU were aligned to the sliced alignment with PyNAST (72) with default parameters. The alignment was filtered with filter_alignment.py script in Qiime (73) using default parameters. Phylogeny was inferred with FastTree 2 (74).

### Statistical analyses.

Sample metadata, OTU table, phylogenetic tree, taxonomic assignments, and representative OTU sequences were imported as a phyloseq (75) object into R (76). All downstream analyses were conducted in R and plotted with ggplot2 (77). Tables S1 to S6 are available at the GitHub repository at https://github.com/ukaraoz/BiocrustSuccessionWetup.

### Phylogenetic structure analysis.

We used the abundance-weighted mean pairwise distances (“mpd” in Picante) to calculate the net relatedness index (NRI [78]) and net taxon index (NTI [78]) for microbial communities in each crust maturity level in each replicate subplot pre- and postwetting. NRI and NTI are standardized metrics of phylogenetic relatedness describing whether an observed community is a phylogenetically biased subset of the taxa that could coexist in the source pool. NRI is calculated as mean pairwise distance (MPD) between all OTUs, as measured by the branch lengths, in an observed sample compared to random draws from the OTU pool (36, 78). NTI is similarly calculated for the nearest relatives based on the terminal branch lengths and as such is much more sensitive to uncertainty in terminal-level tree resolution (79, 80). Positive values of NRI/NTI are indicative of phylogenetic clustering, while negative values are indicative of phylogenetic evenness (overdispersion). A multitude of null models specifying how random draws of the communities from the taxa pool are performed can be used in calculating NRI/NTI. The choice of null models for significance testing of NRI and NTI affects type I and type II error rates (81, 82). In this study, we used the phylogeny shuffle model (“taxa.labels” in the Picante package in R [83]), which shuffles taxon labels across the phylogeny while keeping the phylogenetic relationships intact, hence fixing the total abundance of taxa within and across communities, the occurrence frequencies of taxa, taxa alpha and beta diversity, and patterns of spatial contagion of taxa.

### Estimation of 16S gene copy numbers for OTUs.

rRNA gene operon copy numbers were estimated at the OTU level using available whole-genome-sequenced representatives of OTU taxonomy. For each OTU, the most specific taxonomic classification was used to lookup for the mean number of operons in *rrn*DB version 5.0 (84). The *rrn*DB estimate tool was used to adjust for 16S copy number variation.

### Phylogenetic placement of wetting-responsive OTUs.

For wetting-responsive OTUs, we repeated the phylogeny estimations within their respective families using a more accurate but slow algorithm (RAxML) (85). For each noncyanobacterial wetting-responsive OTU (Table 1), 16S rRNA sequences from their associated families were downloaded from the NCBI taxonomy database (86) and aligned together with the OTU representative sequence using MUSCLE (87) with default parameters. Alignments were trimmed to cover V4 region. Phylogeny of OTUs was inferred by RAxML using the GTRGAMMA model with 500 bootstrapped replicates. RAxML was called as follows: *raxmlHPC-PTHREADS-SSE3 -# 500 -m GTRGAMMA -p 777 -x 2000 -f an -s inputalignment*. *V4 -n outputtree -T 4*.

### Network analysis.

Rare OTUs are prone to cause artifacts in the network analysis (23, 88). In order to avoid spurious correlations, we first removed OTUs with a maximum relative abundance below 0.5% of the total number of reads across all samples. Co-occurrence metrics were estimated using SparCC (26) based on rRNA gene relative abundances in dry and wet (25.5 h after wetting) samples. OTU pairs with SparCC correlations with absolute values of ≥0.3 were considered to exhibit a co-occurrence relationship. Co-occurrence patterns were visualized using Cytoscape (89) as an undirected graph in which each OTU and co-occurrence was indicated by a node and edge, respectively.

### Accession number(s).

The SSU rRNA gene and rRNA sequences have been deposited in the NCBI Bioproject database under accession no. PRJNA299730.
