# Supplementary figures and images for: Large Blooms of Bacillales (Firmicutes) Underlie the Response to Wetting of Cyanobacterial Biocrusts at Various Stages of Maturity
Source: mBio. 2018 Mar 6;9(2):e01366-16. doi: 10.1128/mBio.01366-16 (PMC5844995; doi:10.1128/mBio.01366-16)

Figure S1

a.

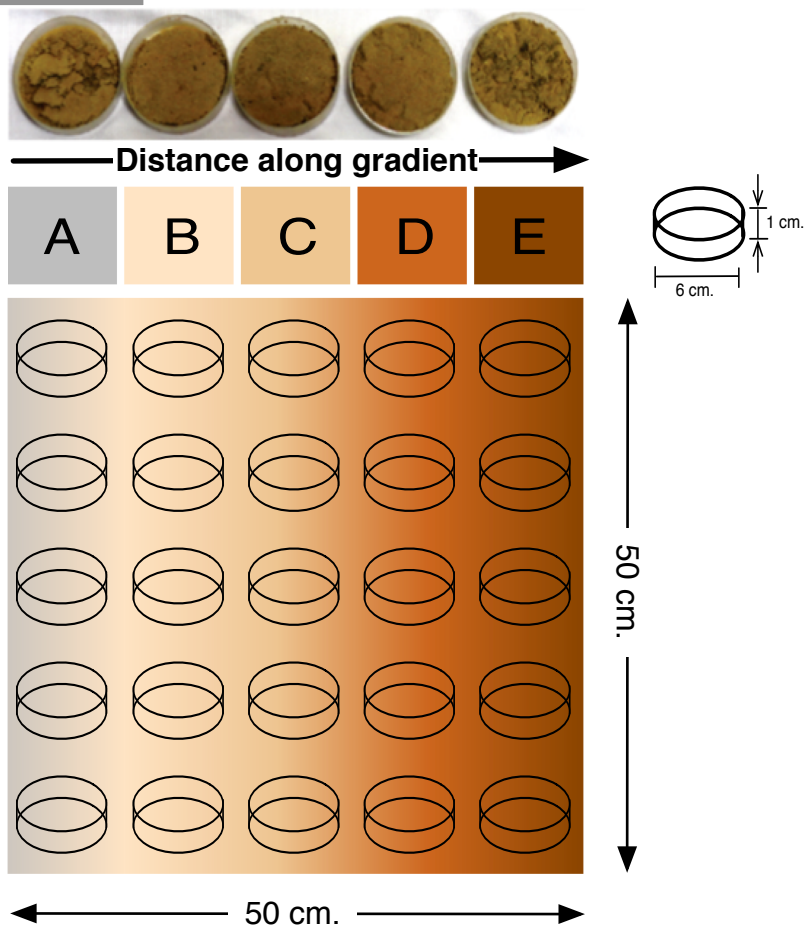

b.

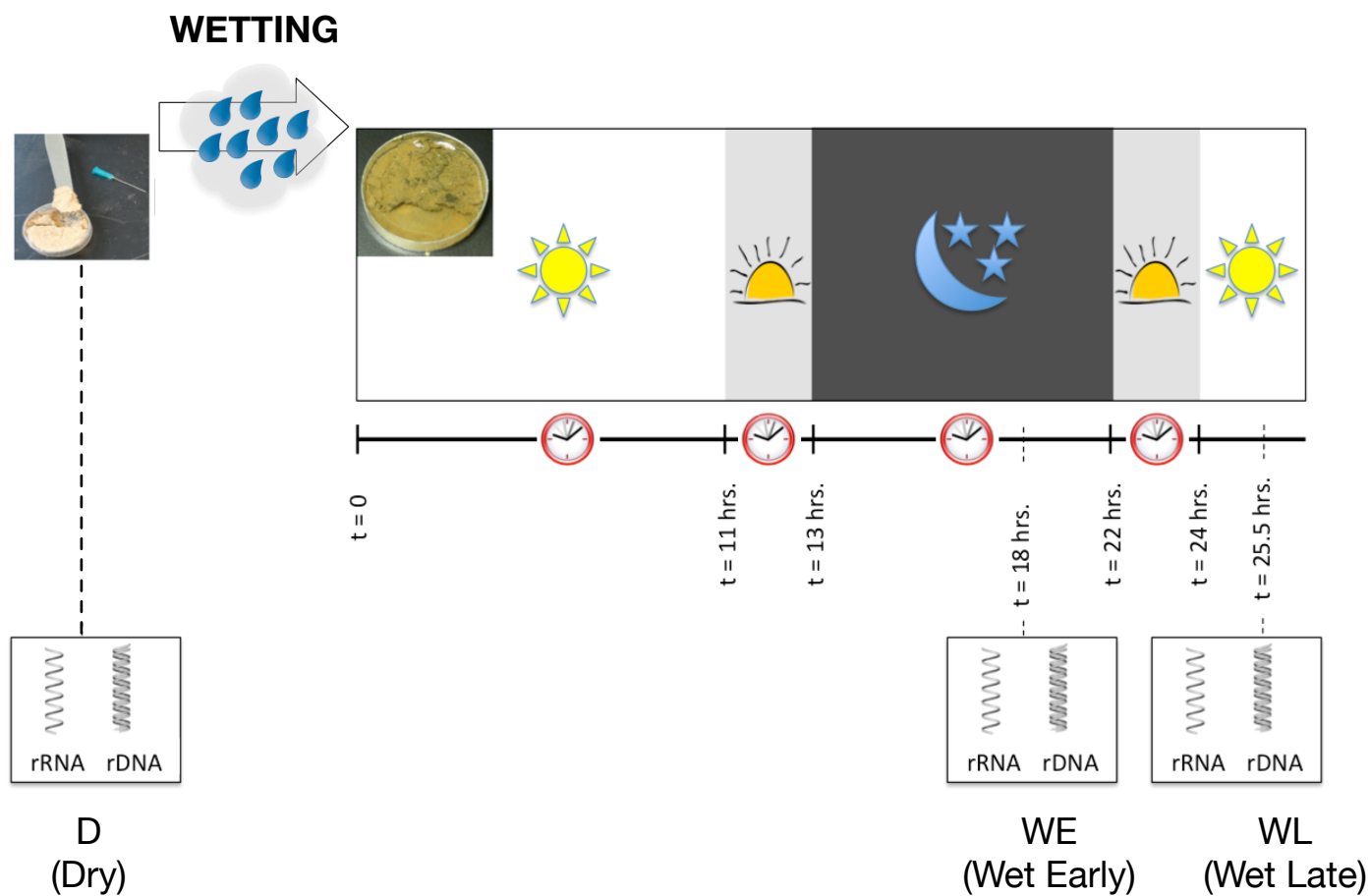

Supplement: FIG S1 [file mbo001183751sf1.pdf]

Figure S2

a.

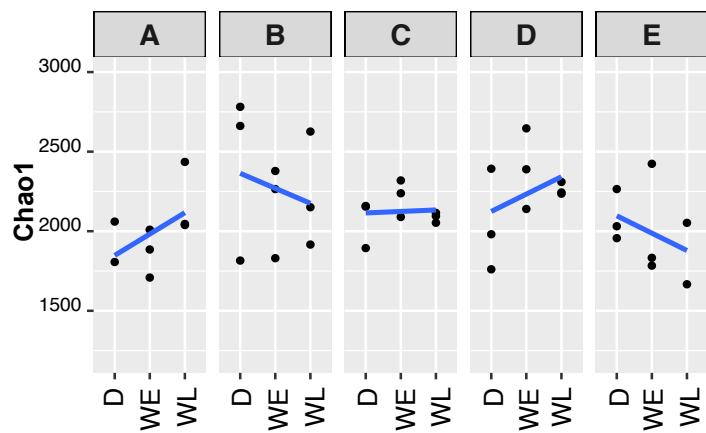

b.

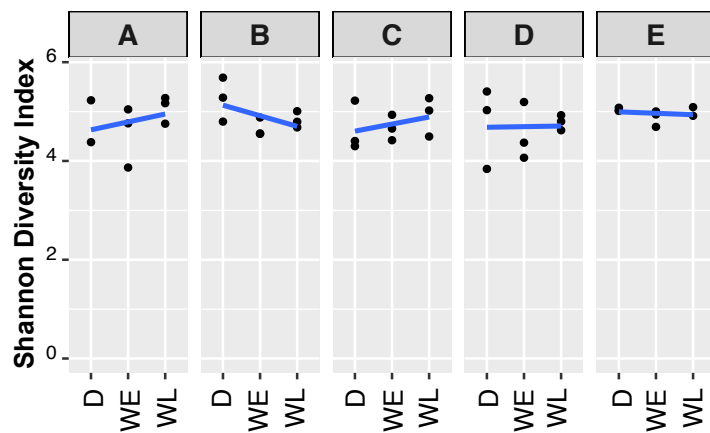

c.

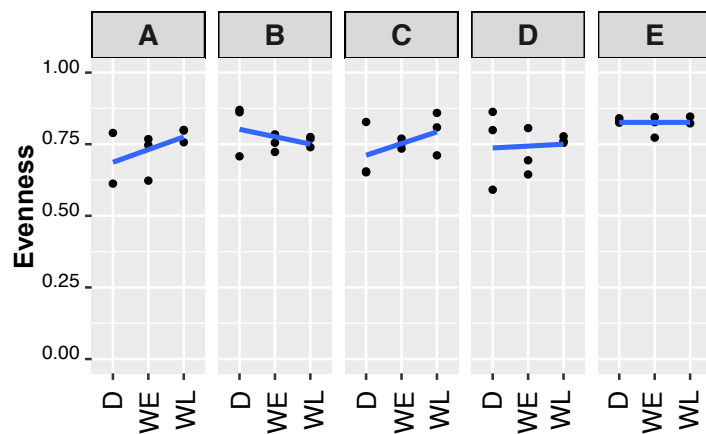

d.

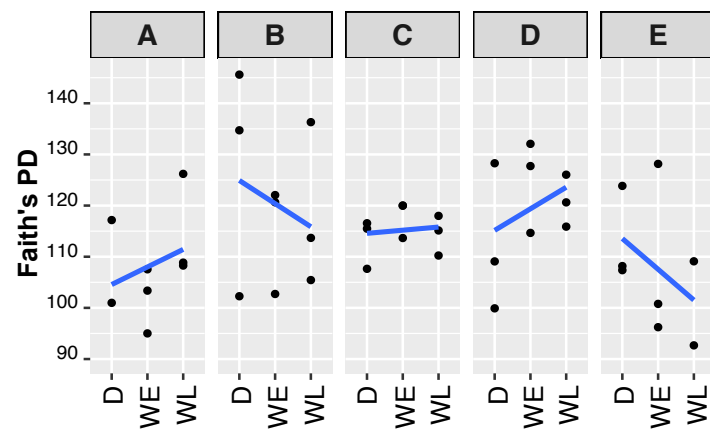

e.

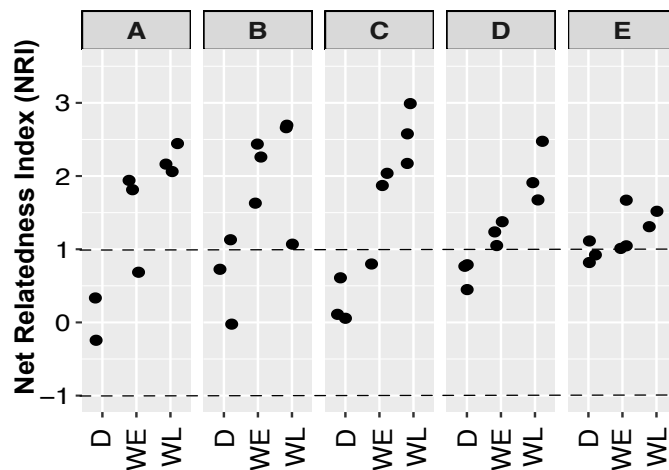

f.

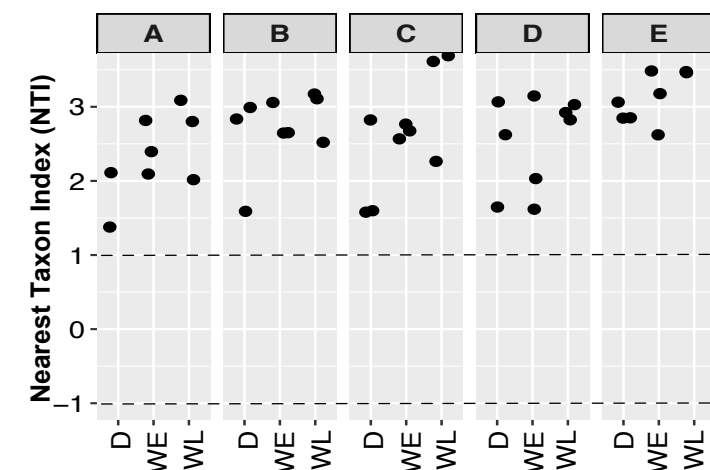

Supplement: FIG S2 [file mbo001183751sf2.pdf]

Figure S3

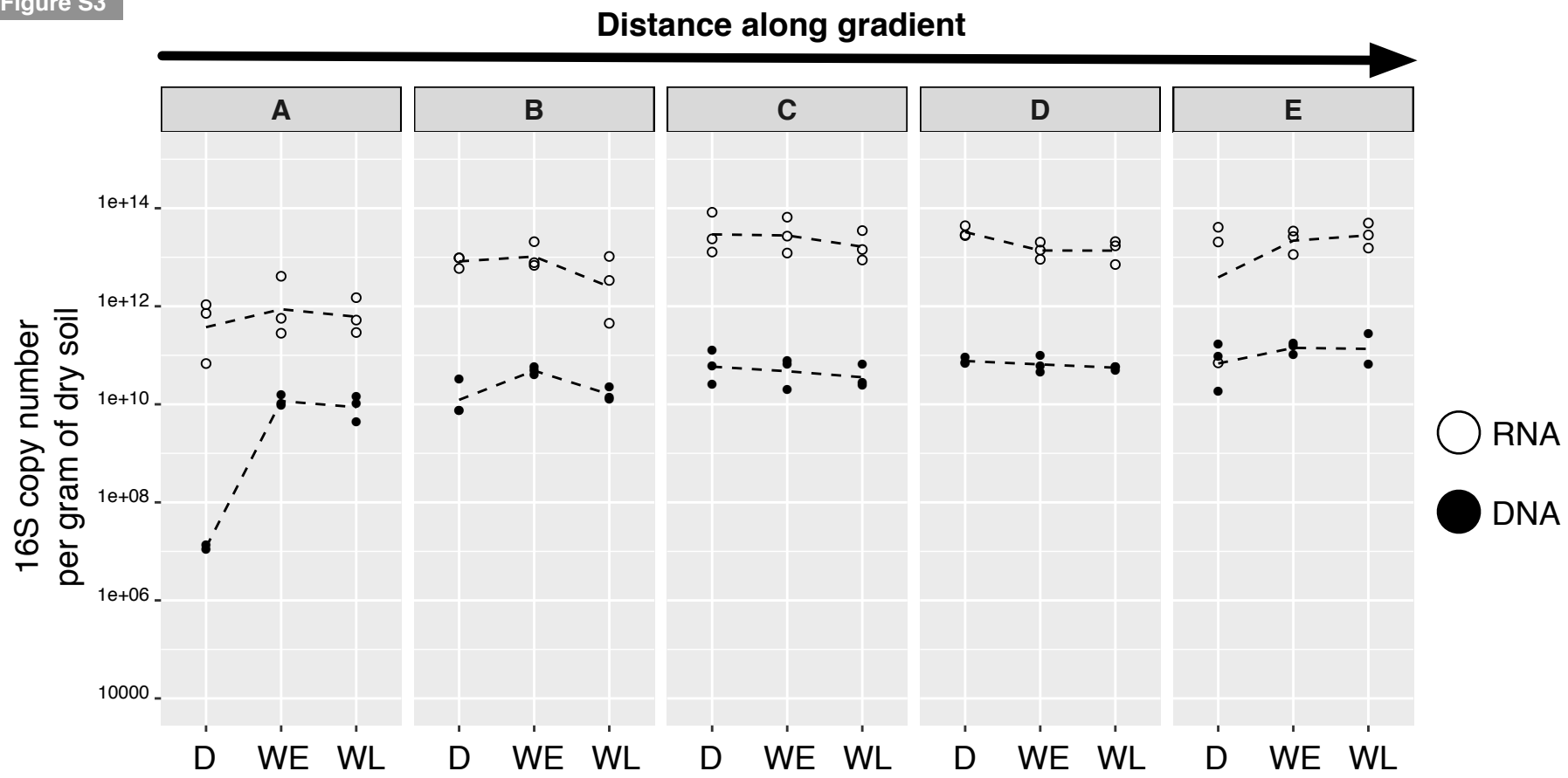

Supplement: FIG S3 [file mbo001183751sf3.pdf]

Figure S4

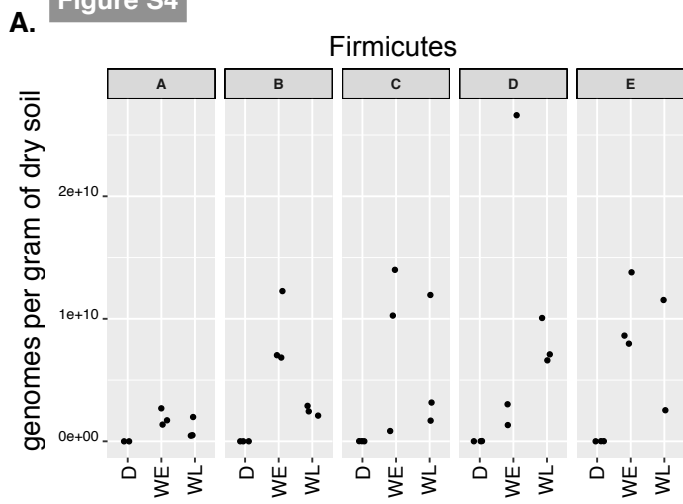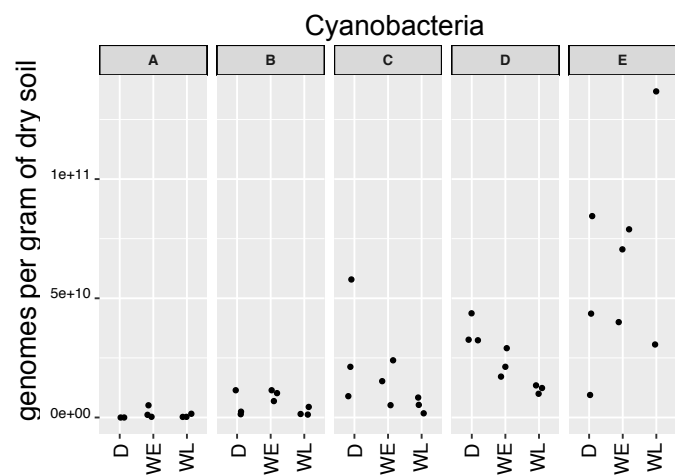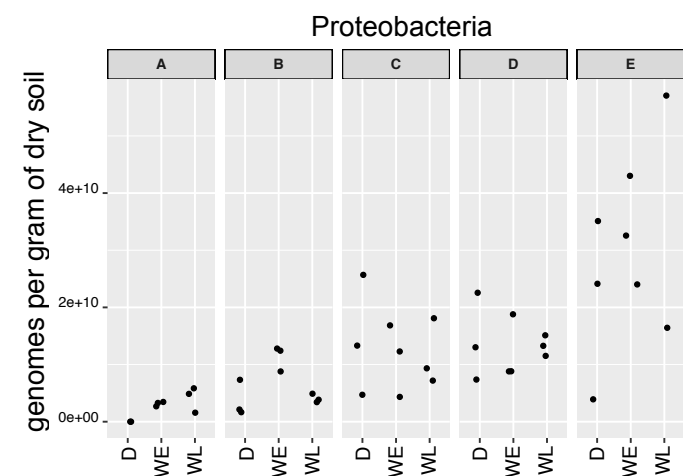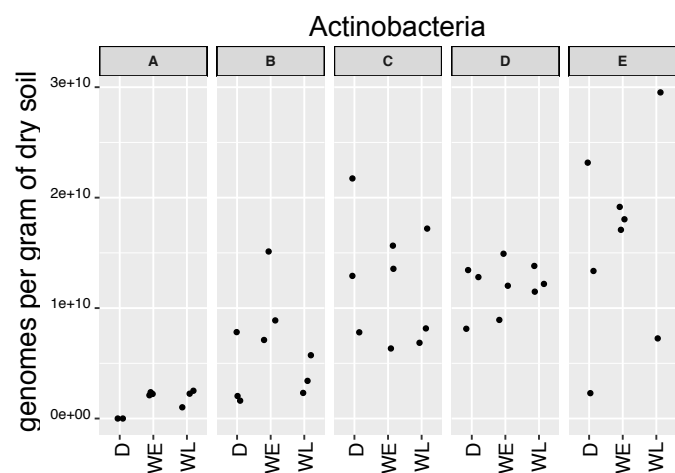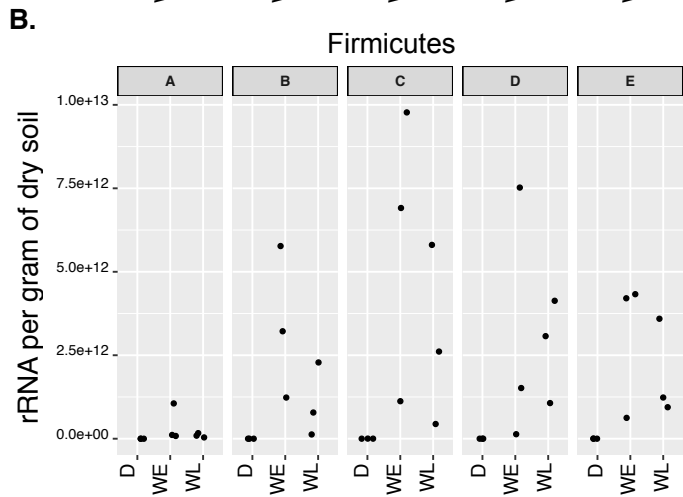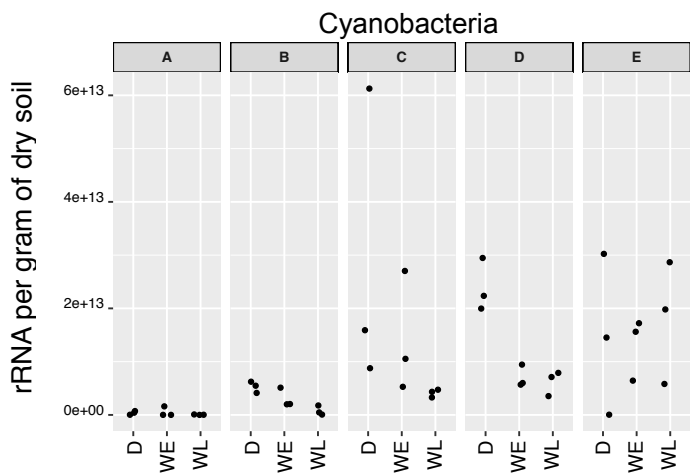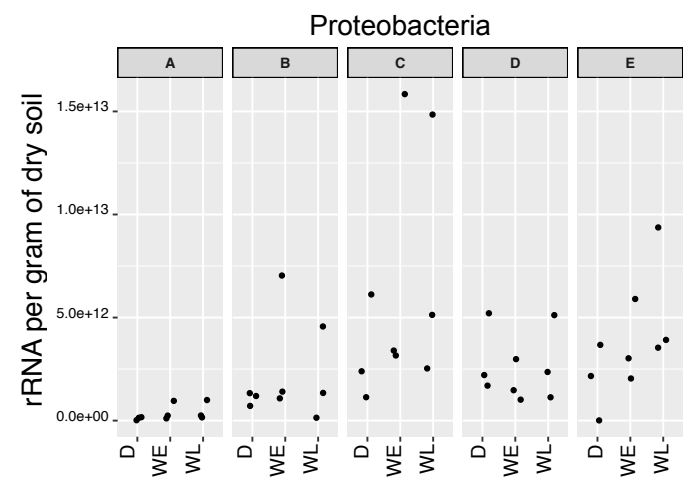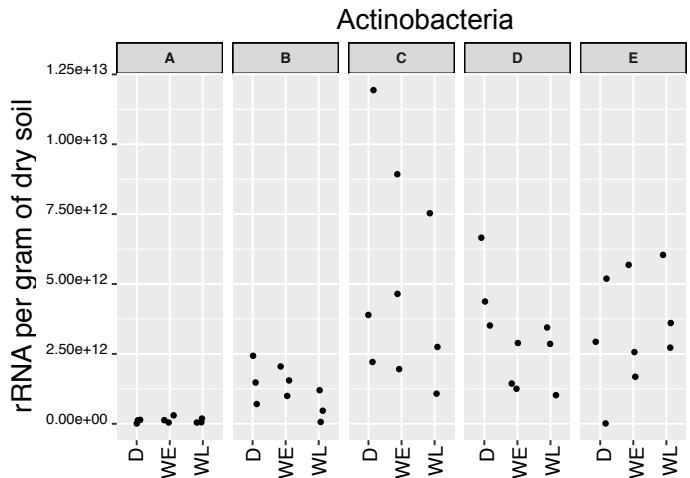

Supplement: FIG S4 [file mbo001183751sf4.pdf]

**A.** Figure S5

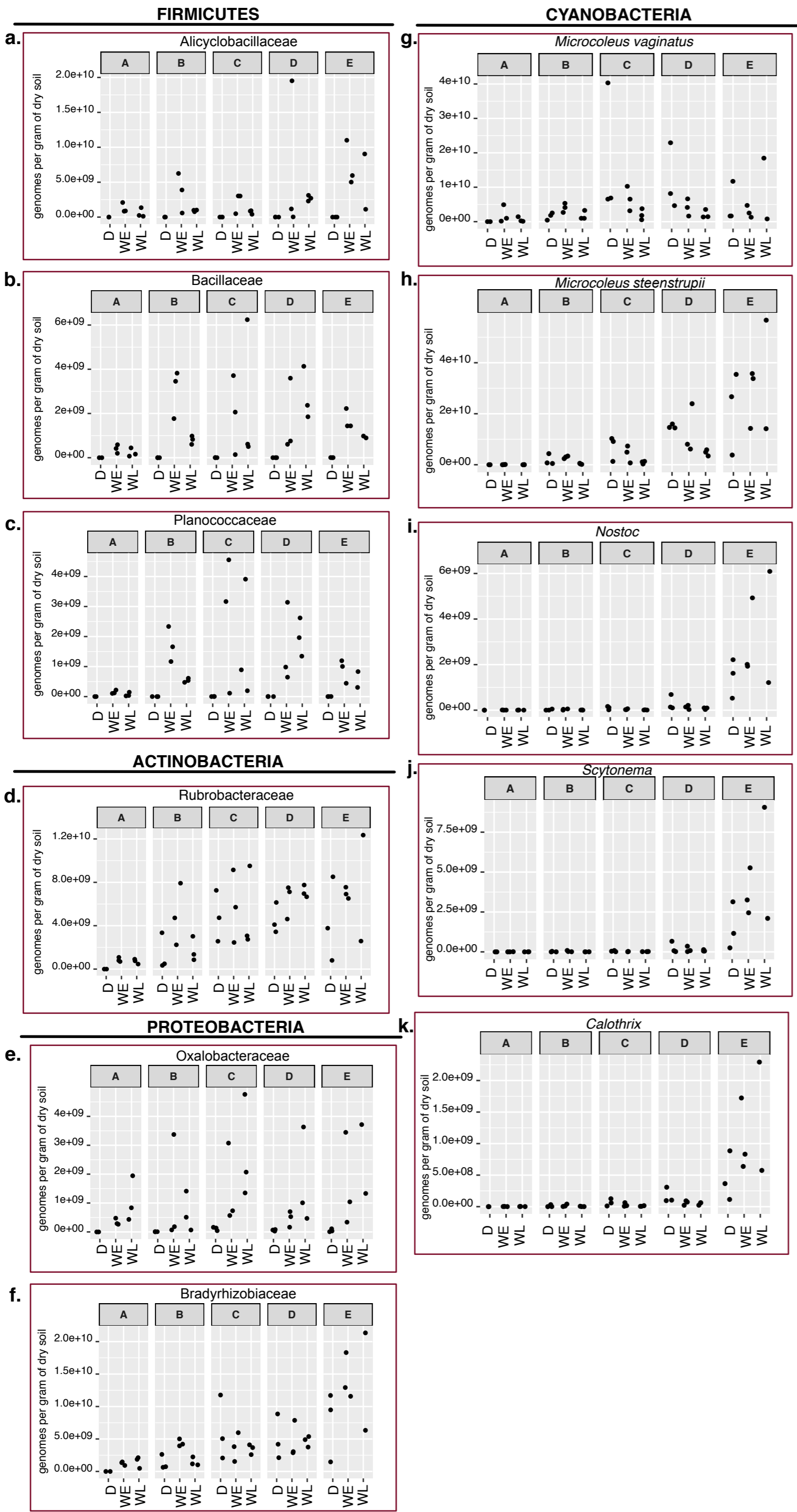

**B.**

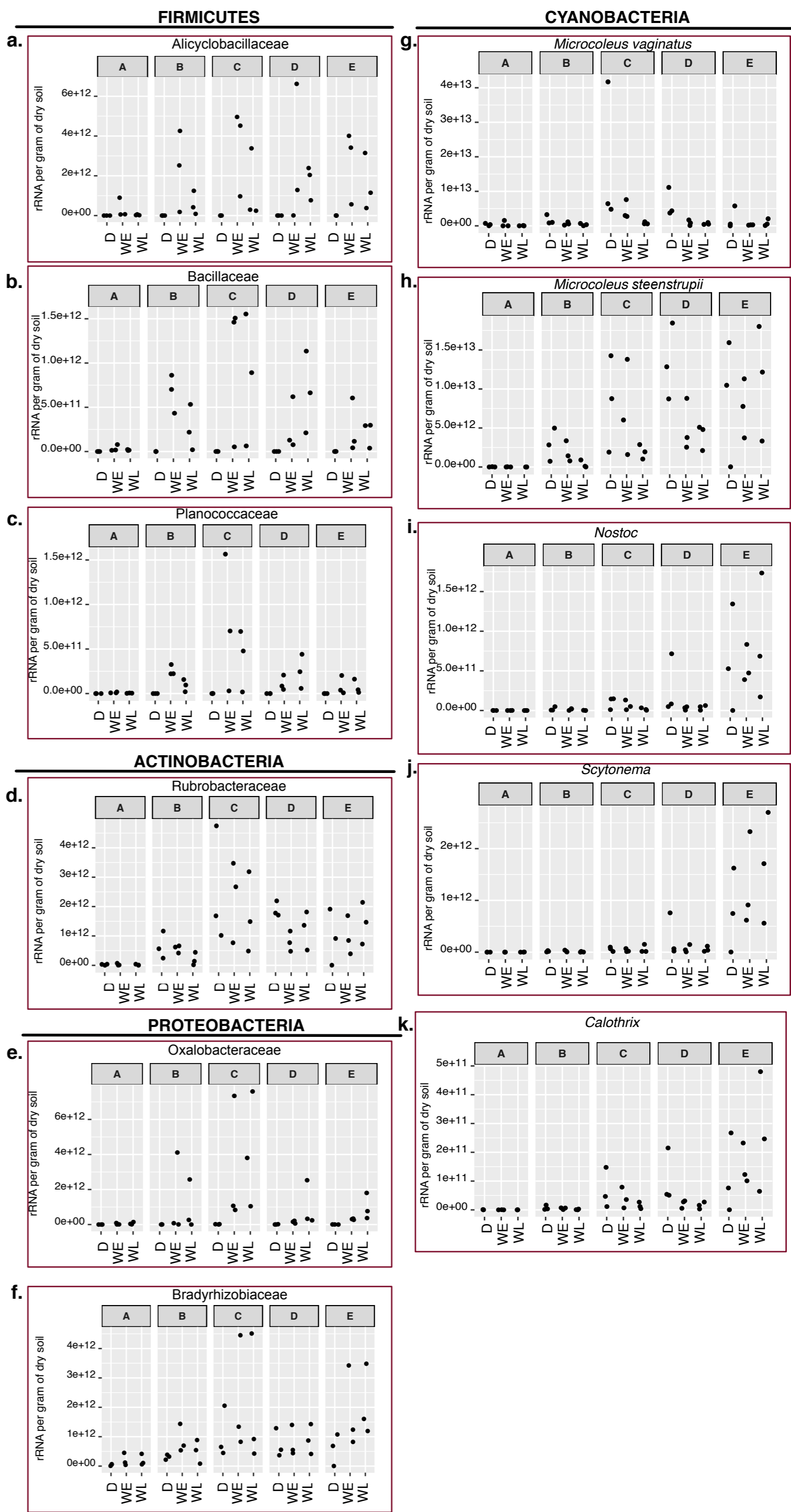

Supplement: FIG S5 [file mbo001183751sf5.pdf]

Figure S6

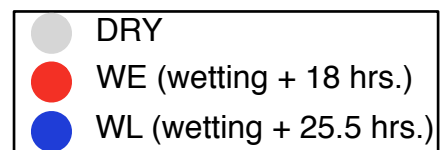

Distance along gradient

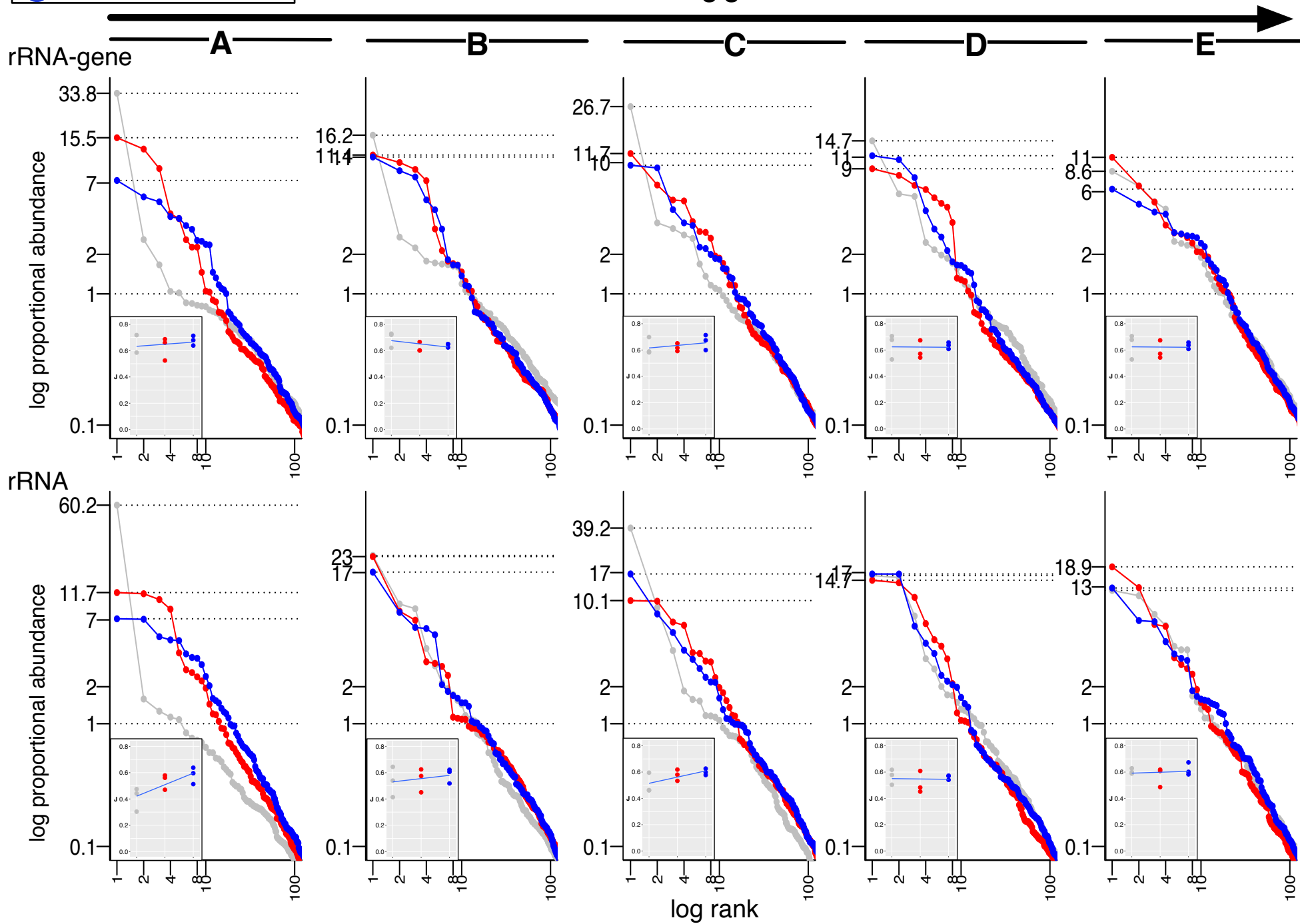

Supplement: FIG S6 [file mbo001183751sf6.pdf]

Figure S9

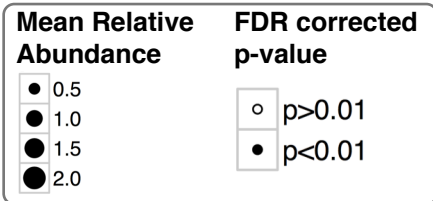Actinobacteria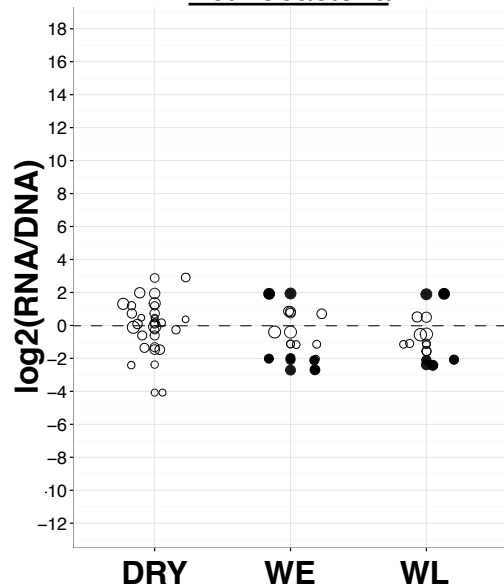Cyanobacteria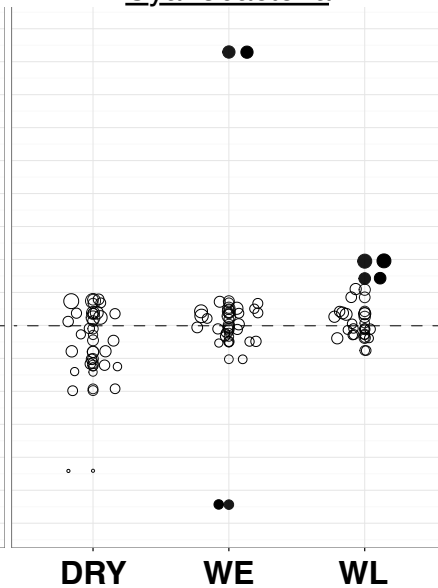Proteobacteria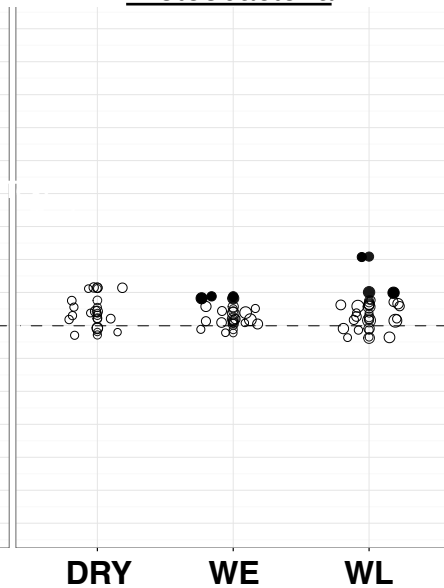Firmicutes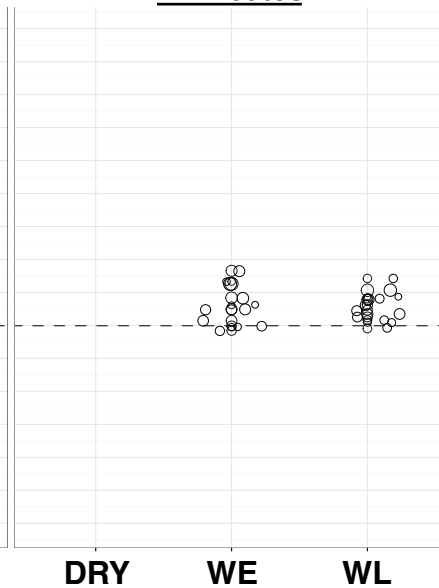

Supplement: FIG S9 [file mbo001183751sf9.pdf]

Figure S10

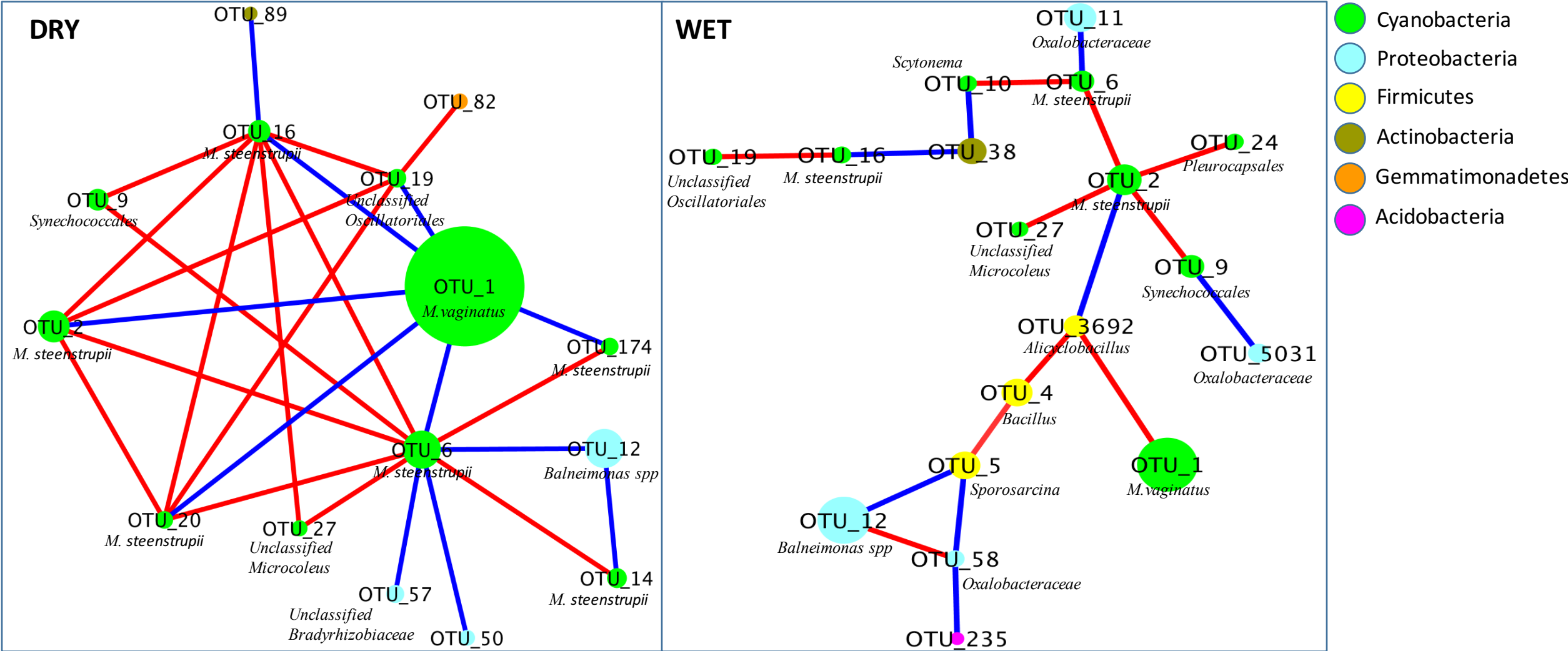

Supplement: FIG S10 [file mbo001183751sf10.pdf]
