# Supplementary material for: Large Blooms of Bacillales (Firmicutes) Underlie the Response to Wetting of Cyanobacterial Biocrusts at Various Stages of Maturity
Source: mBio. 2018 Mar 6;9(2):e01366-16. doi: 10.1128/mBio.01366-16 (PMC5844995; doi:10.1128/mBio.01366-16)
Supplement: FIG S7 [file mbo001183751sf7.pdf]

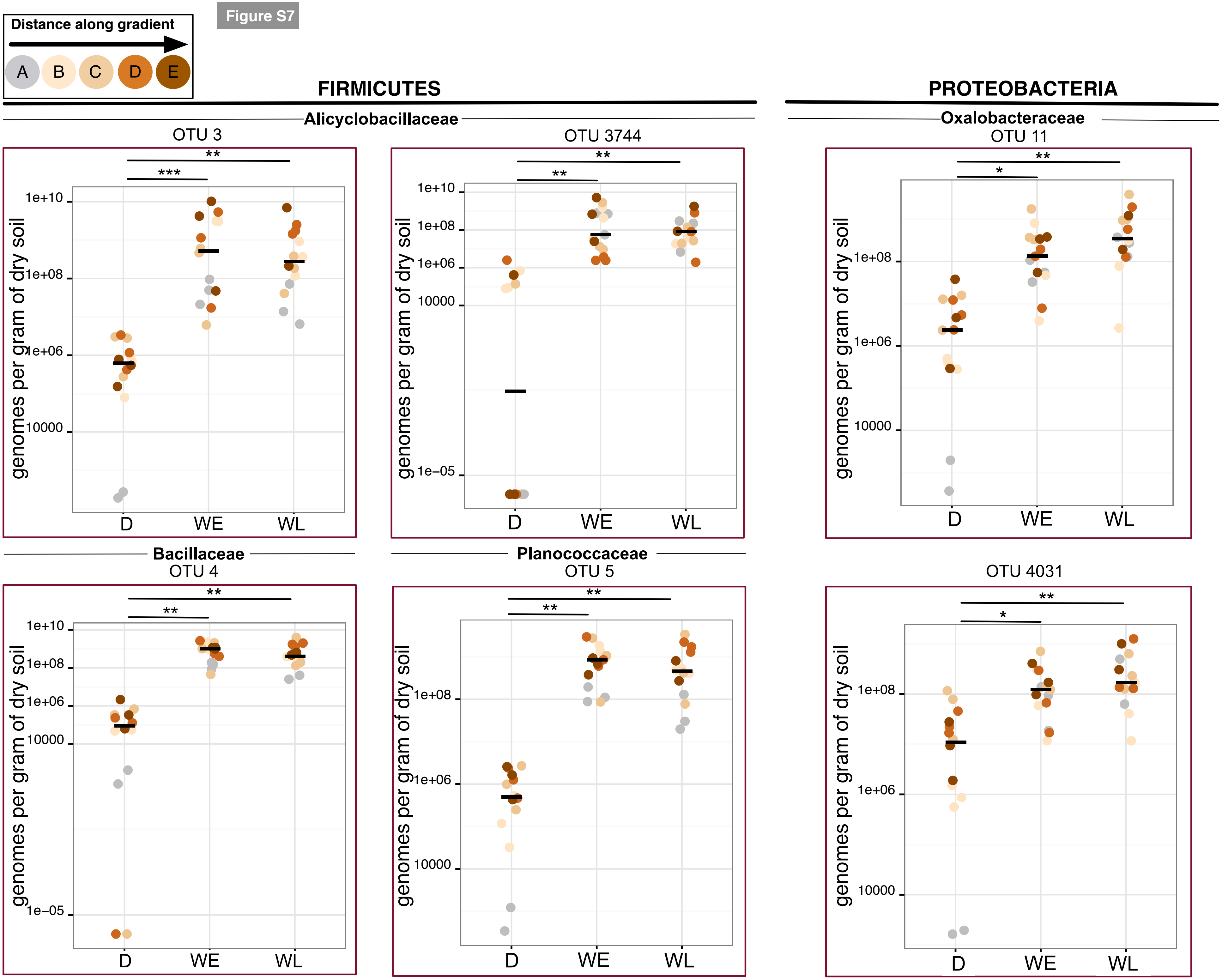
Figure S7
Distance along gradient
A B C D E
FIRMICUTES
PROTEOBACTERIA
Alicyclobacillaceae
OTU 3
OTU 3744
Oxalobacteraceae
OTU 11
Bacillaceae
OTU 4
Planococcaceae
OTU 5
OTU 4031
genomes per gram of dry soil
1e+10
1e+08
1e+06
10000
1e−05
D
WE
WL
*
**
***
