# Supplementary material for: Large Blooms of Bacillales (Firmicutes) Underlie the Response to Wetting of Cyanobacterial Biocrusts at Various Stages of Maturity
Source: mBio. 2018 Mar 6;9(2):e01366-16. doi: 10.1128/mBio.01366-16 (PMC5844995; doi:10.1128/mBio.01366-16)

Figure S8

a. Family *Alicyclobacillaceae* (Firmicutes)

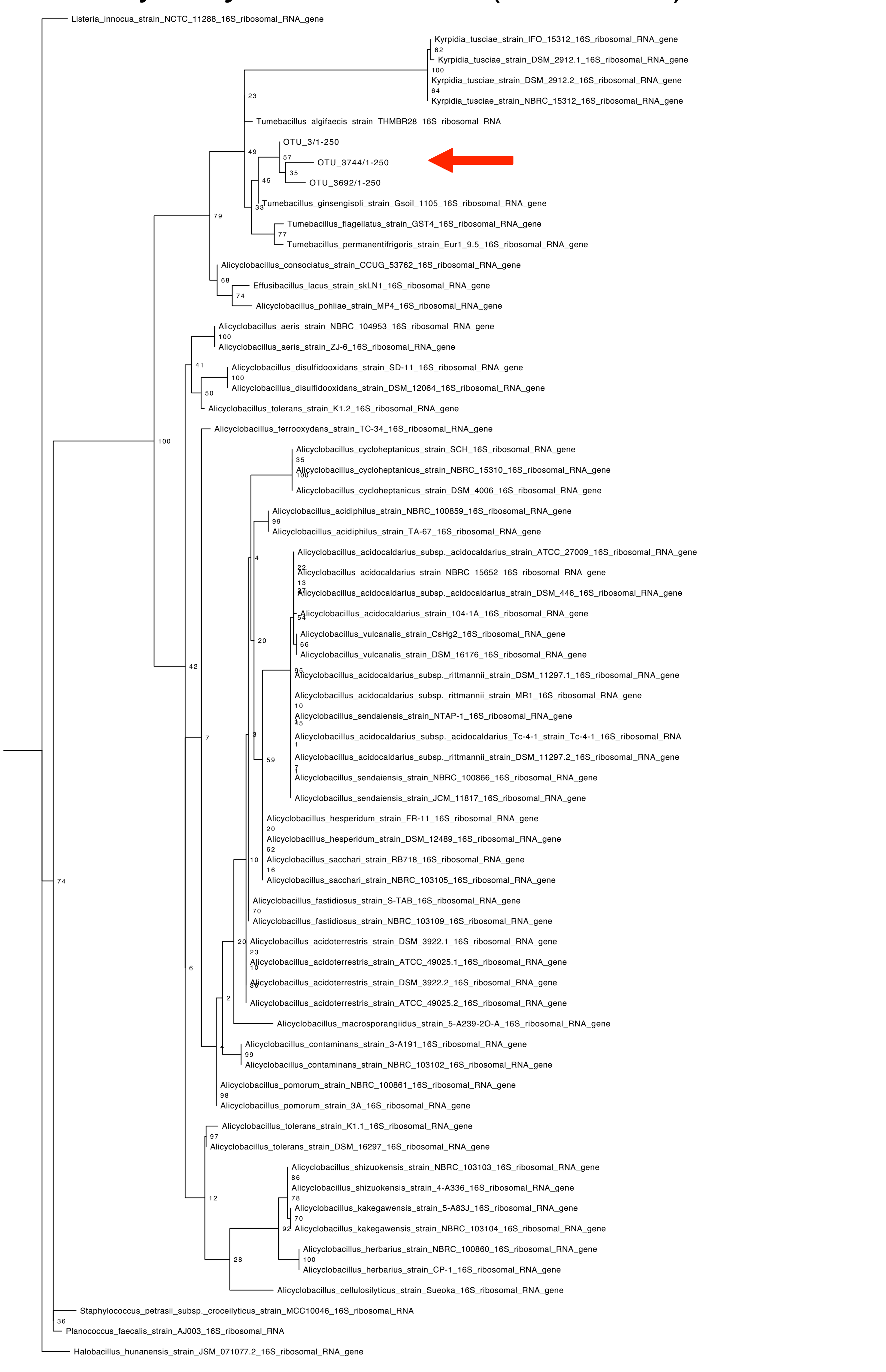

b. Family *Bacillaceae* (Firmicutes)

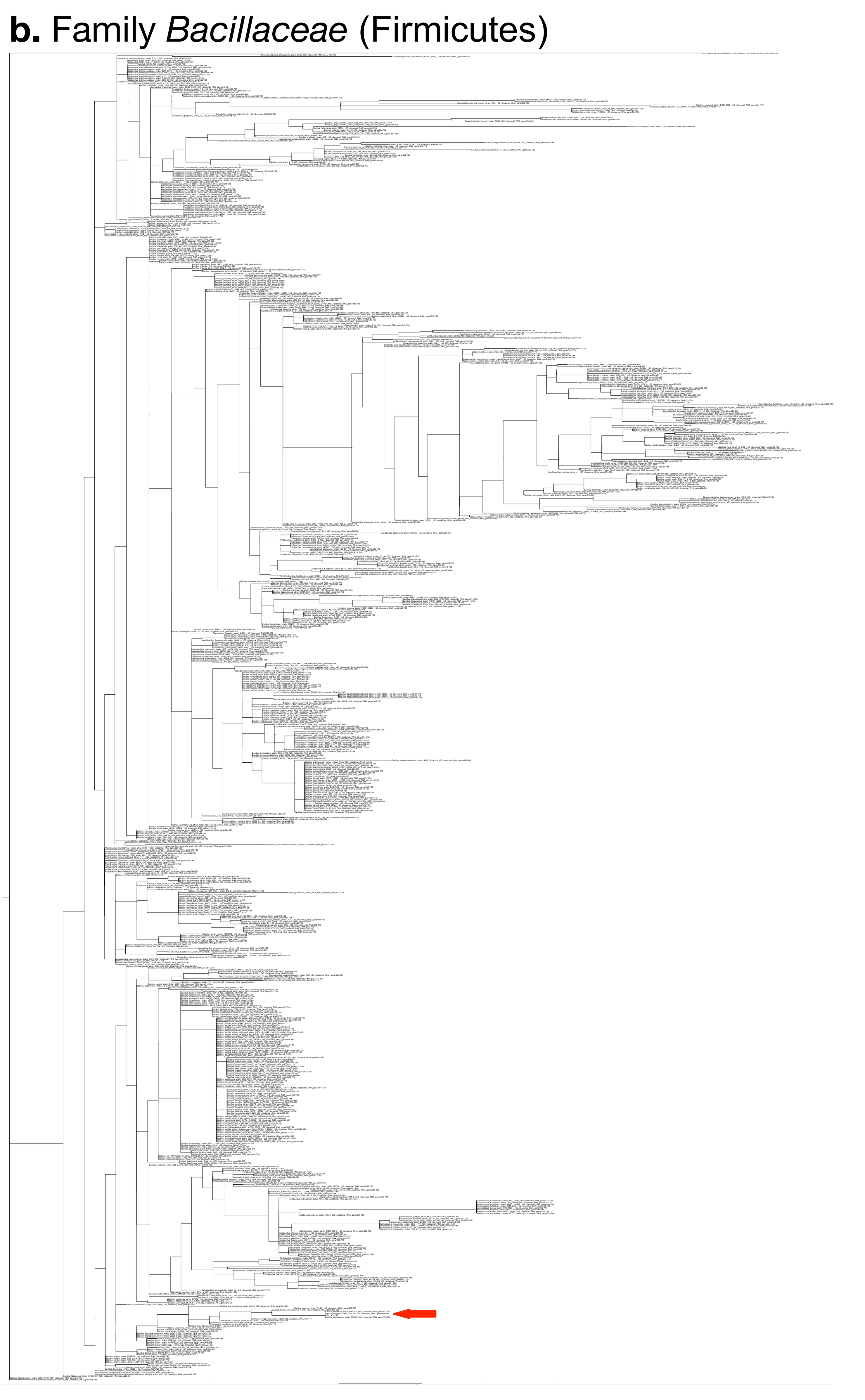

c. Family *Planococcaceae* (Firmicutes)

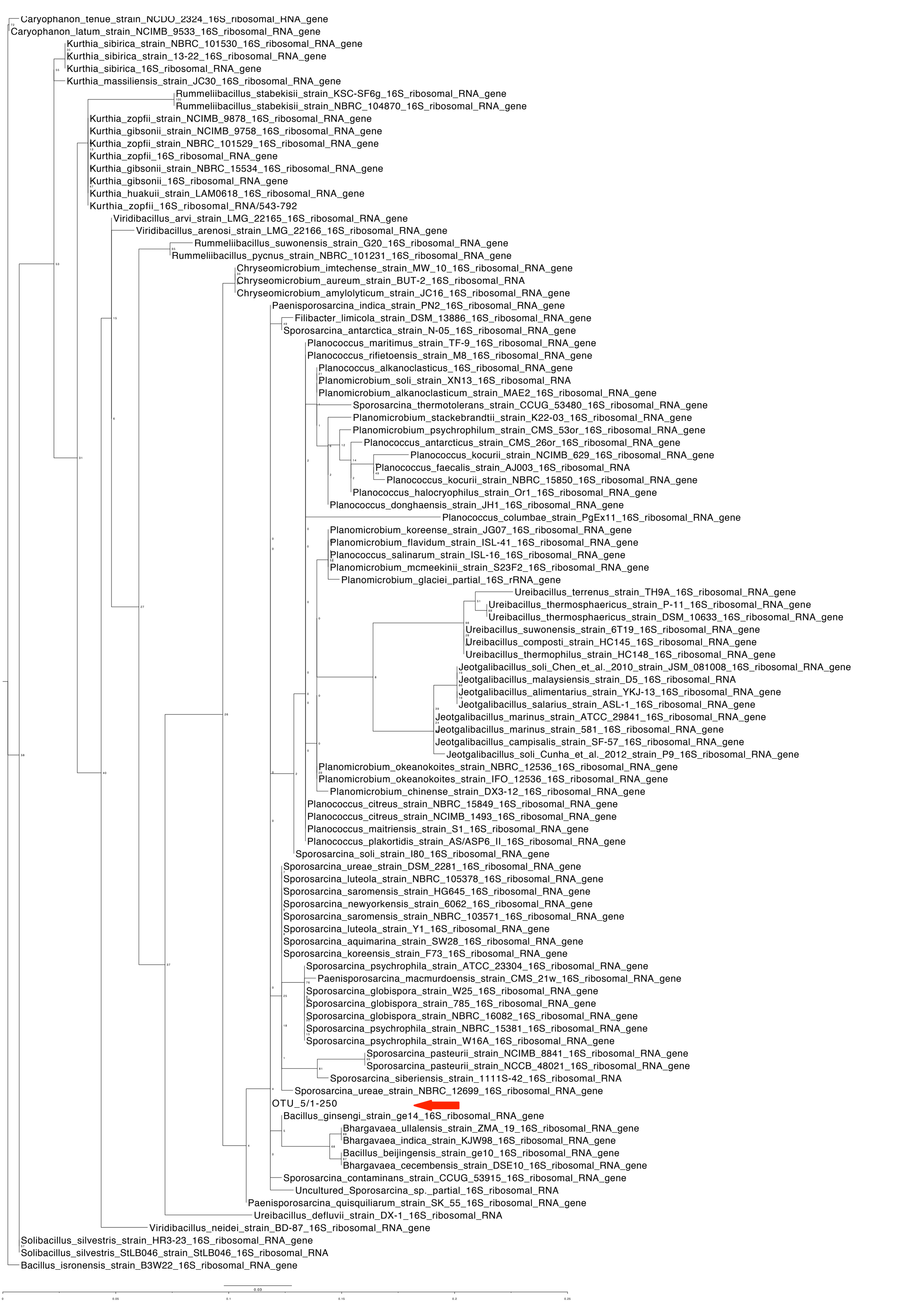

d. Family *Rubrobacteraceae* (Actinobacteria)

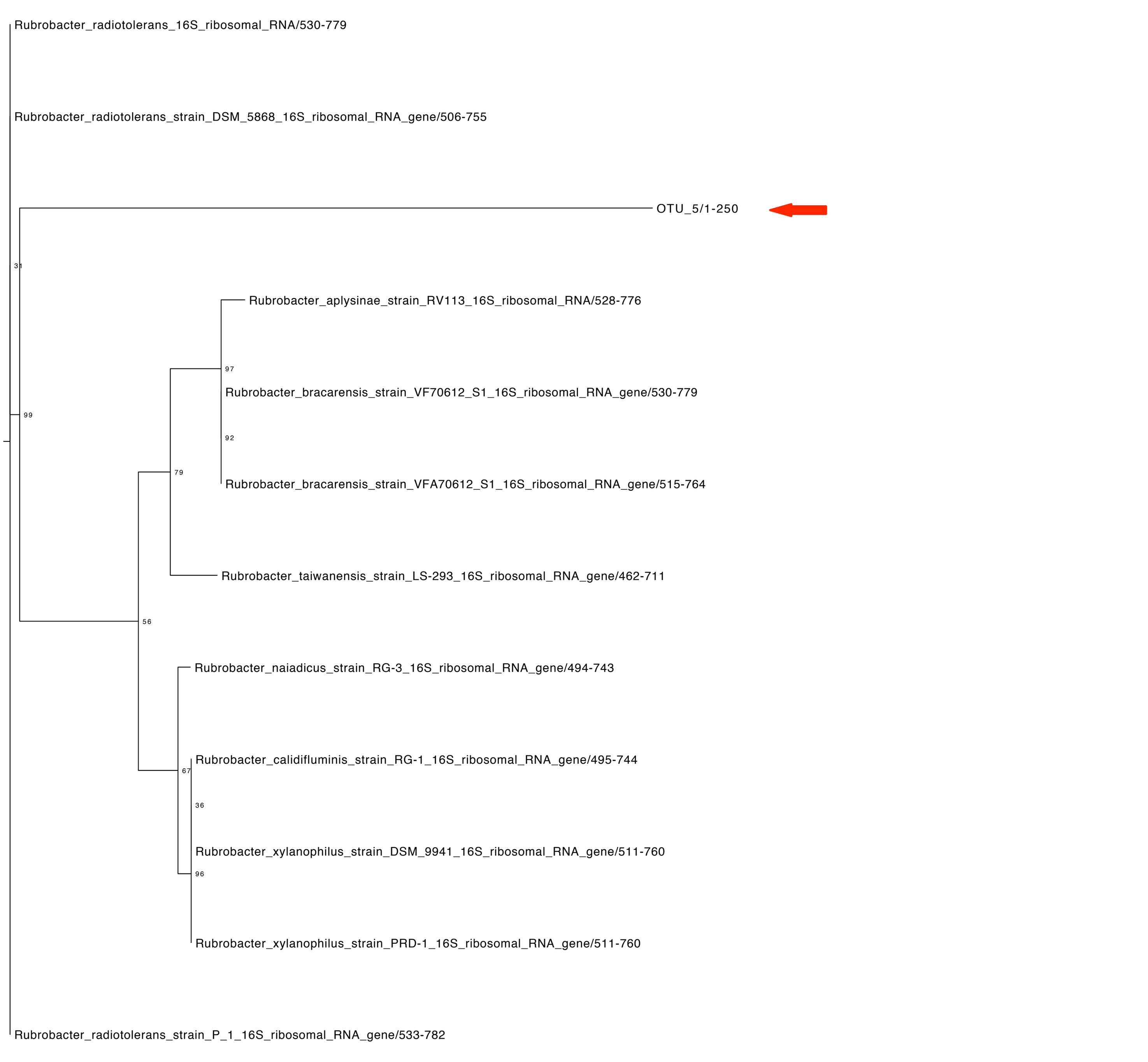

e. Family *Bradyrhizobiaceae* (Proteobacteria)

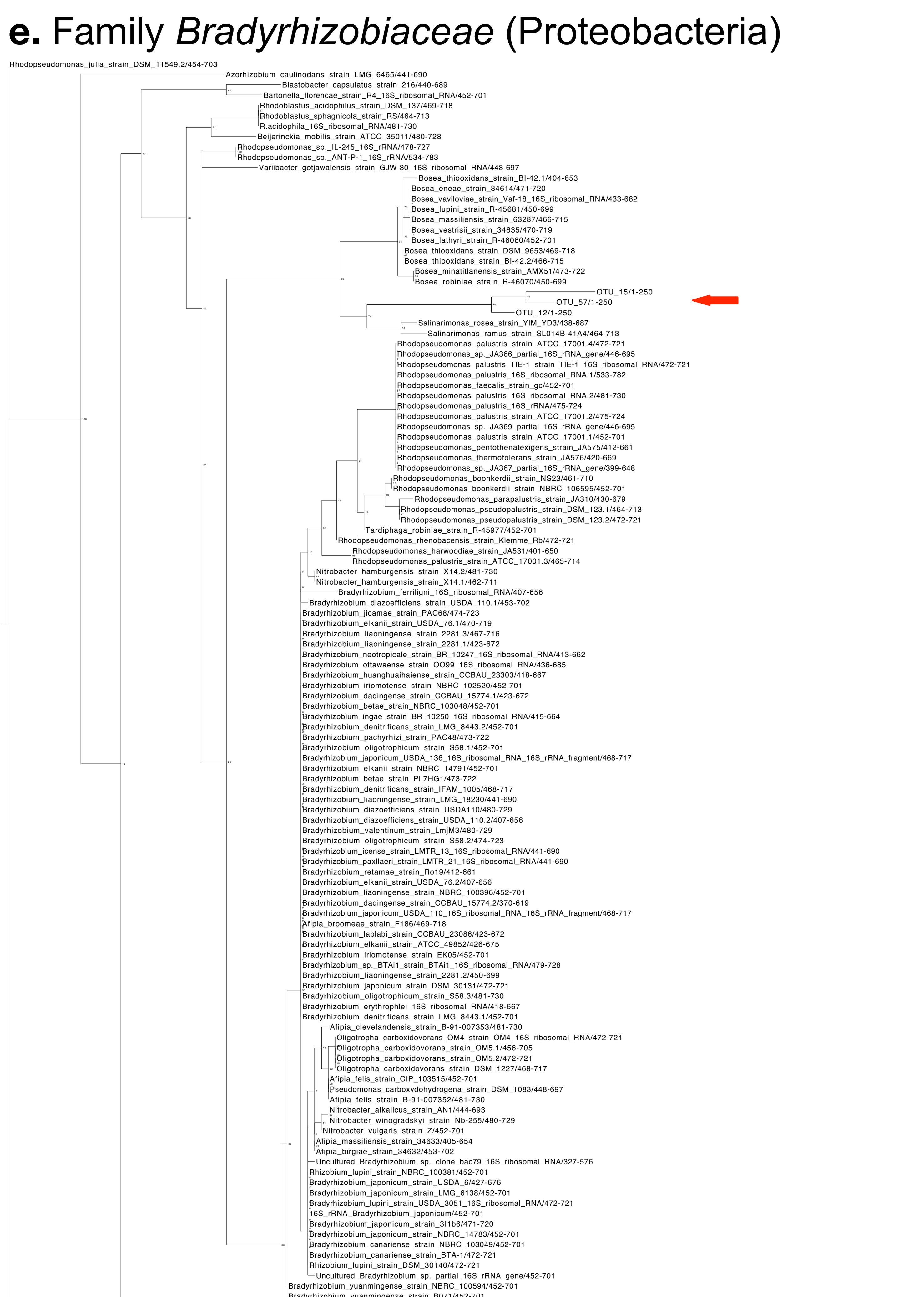

f. Family *Oxalobacteraceae* (Proteobacteria)

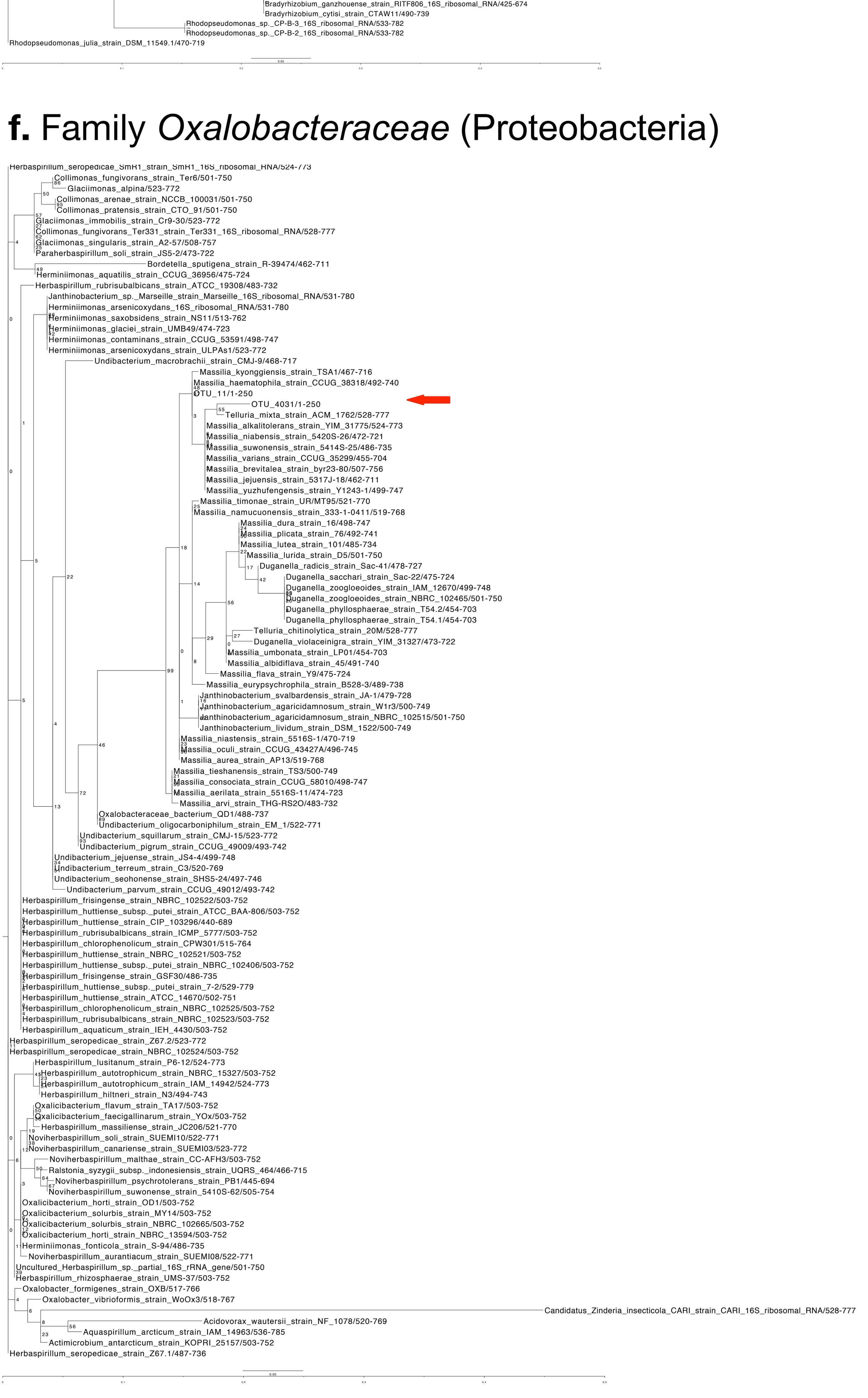

Supplement: FIG S8 [file mbo001183751sf8.pdf]
